# Developing attentional control in naturalistic dynamic road crossing situations

**DOI:** 10.1038/s41598-019-39737-7

**Published:** 2019-03-12

**Authors:** Victoria I. Nicholls, Geraldine Jean-Charles, Junpeng Lao, Peter de Lissa, Roberto Caldara, Sebastien Miellet

**Affiliations:** 1 Bournemouth University, Faculty of Science & Technology, Bournemouth, BH12 1PH United Kingdom; 2 University of Fribourg, Department of Psychology, iBM lab, Fribourg, 1700 Switzerland; 3 University of Wollongong, School of Psychology, ActiveVision lab, Wollongong, 2522 Australia

## Abstract

In the last 20 years, there has been increasing interest in studying visual attentional processes under more natural conditions. In the present study, we propose to determine the critical age at which children show similar to adult performance and attentional control in a visually guided task; in a naturalistic dynamic and socially relevant context: road crossing. We monitored visual exploration and crossing decisions in adults and children aged between 5 and 15 while they watched road traffic videos containing a range of traffic densities with or without pedestrians. 5–10 year old (y/o) children showed less systematic gaze patterns. More specifically, adults and 11–15 y/o children look mainly at the vehicles’ appearing point, which is an optimal location to sample diagnostic information for the task. In contrast, 5–10 y/os look more at socially relevant stimuli and attend to moving vehicles further down the trajectory when the traffic density is high. Critically, 5-10 y/o children also make an increased number of crossing decisions compared to 11–15 y/os and adults. Our findings reveal a critical shift around 10 y/o in attentional control and crossing decisions in a road crossing task.

## Introduction

Human visually guided behaviour relies on the selective uptake of information, due to sensory and cognitive limitations^1^. In other words, human vision is a dynamic process, during which the observer actively samples the environment in order to gather diagnostic information for the task at hand. This is made possible by our attentional systems selecting information based on bottom-up stimulation^2–6^ and top-down influences^7–11^.

The focus on top-down processes has been of increasing interest in the last 20 years, as the study of visual processing has sought to involve more natural conditions and realistic stimuli. Many recent studies used photographs and included eye movement recordings to look at the influence of context on visual attention^11–16^. For instance, these studies have shown influences of semantic^17^, episodic top-down processes^18–20^, as well as scene context on parafoveal processing of objects^21,22^. More recently, the role of the observer’s intention and understanding of the scene has been emphasized^23,24^. In this perspective, oculomotor planning is seen as making predictions about the locations of diagnostic information for the task^25^.

These top-down processes are generally considered as being mainly under control of the frontal regions, which are still maturing during childhood^26–31^. This protracted maturation of the frontal lobes has been associated with a lack of top-down attentional control^32^ and a deficit of top-down inhibition of reflexive, automatic saccades^33,34^. This is consistent with research showing more express saccades, a slower pro- and anti-saccade reaction time and a higher error rate in the anti-saccade tasks for children compared to adults^35–38^. In terms of critical age, Leclercq and Siéroff^39^ suggested that 6 and 8 (y/o) year old children fail to inhibit attentional capture by goal irrelevant stimuli while 10 y/os and adults succeed. Klein,Fischer, Hartnegg, Heiss and Roth^37^; Klein and Foerster^36^ showed that adults exhibited faster saccades and fewer prosaccades during the anti-saccade task than 10–11 y/os, who in turn had faster sacacdes and fewer prosaccades than 6 and 7 y/os. Additionally, Munoz and colleagues^38^ showed that children between 5 and 8 y/o had slow saccadic reaction times (SRTs) and the most direction errors in the anti-saccade task, which is related to the protracted maturation of the frontal lobes^33^. These studies suggest that children between 8 and 10 y/o have similar attentional control skills to adults. However, it is important to mention that most of these studies used basic situations involving only flashing of very simple target stimuli. As explained above, more naturalistic situations and stimuli involve top-down processes more strongly. Thus, it is possible that the complexity and realism of the task influences the critical age at which children show stronger oculomotor capture and decreased inhibition of responses to task irrelevant distractors compared to adults. Using static natural images, Açık and colleagues^40^ found that children under the age of 10 use oculomotor strategies particularly influenced by bottom-up processes. These authors tested children from 7–9 y/o, adults from 19–27 y/o, and older adults above 72 years of age. More recently, Kuhn and Teszka^41^ explored differences in attentional control between adults and children within a more natural context. Their results suggest that children below the age of 10 are more distracted than adults and this influences how they experience the world around them.

In the present study, we propose to determine the trajectory of the difference in attentional control, its effect on information sampling, and behaviour between children and adults. Moreover, the present study aims to determine whether there is a critical age at which children show similar attentional control skills and behavioural performance to adults, in a naturalistic, dynamic, and socially relevant task. To these aims, we created a road crossing task which requires advanced attentional skills. Crucially, besides addressing an important theoretical question, the present study aims to shed light on a critical practical issue associated with road safety. Road traffic accidents killed 273,000 pedestrians worldwide in 2010 – 22% of all road traffic accidents that year^42^. Regarding child casualties, 186,300 children died from road traffic related incidents across the world in 2012, and 38% of these deaths were child pedestrians^43^. Recurrent observations seem to point towards specific perceptual, cognitive, and behavioural aspects involved in children’s susceptibility to road traffic accidents. For instance, studies using various experimental techniques consistently showed that younger children take longer to enter a safe traffic gap than do older children (judgments on videos^44^, cycling in a virtual environment^45^, road crossing simulation^46^), which overlaps with other developing skills such as perceptual and motor abilities^47^. Previous studies investigating the effect of perceptual processes on road crossing performance reported that children aged under 8 y/o looked less often at traffic^48,49^, and when they did it was often in the opposite direction of oncoming traffic^48^. 8–10 y/os monitored traffic less when vehicles were further away than when they were closer^50^. These looking behaviours were correlated with children under 8–10 y/o making more unsafe crossing decisions.

However, these studies investigated perceptual processes using very general descriptions of the children looking towards or away from the traffic during simulated and real crosswalks^49,51^. Similarly, studies using virtual reality (VR) reported head movements following the traffic^50^ or between computer screens, as well as duration looking at a computer screen^52^. None of these studies, however, provide us with a fine-grained description of how exactly children explore the visual field compared to adults, and how these explorations affect road crossing decisions. Tapiro and colleagues^53^ conducted a more fine-grained analysis of visual attention of adults and children in road crossing situations. Children looked preferentially at areas in front of them, while adults looked preferentially at more distant locations. However, the analysis relies on areas of interest (AOIs) based on a-priori segmentation of the stimulus space, preventing the data driven discovery of meaningful patterns (see^54^ for a discussion on the limitations of AOIs). Critically, to the best of our knowledge, there is no study in the literature that includes distractors and their effects on visual exploration as a way to investigate attention switching and inhibitory control, and how these develop in children for real world scenarios.

The current study aimed to isolate the critical age at which road crossing decisions and oculomotor patterns of children differ from those of adults. We wanted to characterise precisely children’s visual processing specificities and explore the impact of distractors and task complexity. Based on the psychophysics and the road safety literatures our main hypothesis was that children under 10 y/o, for whom studies have shown a reduced inhibitory control attributed to protracted maturation of the frontal lobes^33^ and fewer safe crossing decisions, would produce an increased number of saccades towards task irrelevant stimuli which would, in turn, impact negatively on optimal information sampling for road crossing decisions. We therefore presented child participants aged 5 to 15 (and adult controls) with videos of naturalistic road crossing scenarios. Participants were then asked to decide when to initiate a road crossing and to keep pressing the key as long as the crossing was possible. We included varying levels of traffic density to investigate how this factor influences task difficulty and attention switching, and thus crossing and eye movement behaviours. Additionally, we included pedestrians as they are known to be a potent distractors for attentional capture, in order to test for inhibitory control.

## Results

### Eye Movement Results

#### Global characteristics

General oculomotor characteristics were within a similar range for each age group (see Table 1). Critically, all age groups showed an impact of pedestrians on their global oculomotor characteristics, while only 5–10 y/os showed an impact of traffic density. There was an overall trend for the number of fixations for 5–10 and 11–15 y/os to increase when pedestrians were present in the scene. Adults and 11–15 y/os showed an overall trend to decrease their number of pursuits. All groups showed an overall trend of increasing trial time as fixation when pedestrians were present. Additionally, 11–15 y/os and adults showed an overall trend of decreasing total trial time as pursuit when pedestrians were present. Finally, 5–10 y/os showed an overall trend of decreasing the number of pursuits with lower traffic density. Supplementary Figures S2–S19 and summary S20 provide a detailed description of subtle differences in the distributions at the decile level.

**Table 1:** General oculomotor characteristics. The mean number of and proportion of trial time as each eye movement type. Square brackets contain 95% confidence intervals.

Global characteristics	5–10 yr old	11–15 yr old	Adults
**Fixations**			
Number of	19.15[18.95,19.36]	20.89[20.68,21.11]	21.92[21.58,22.26]
Trial time as(ms)	3319.84[3261,3379]	3635.79[3578,3694]	3435.11[3332,3538]
**Smooth Pursuits**			
Number of	8.79[8.65,8.93]	9.29[9.15,9.43]	10.38[10.11,10.64]
Trial time as(ms)	4738.25[4667,4809]	5043.59[4977,5110]	5164.51[5048,5282]
**Saccades**			
Number of	41.61[40.40,42.80]	39.11[38.52,39.69]	45.02[43.65,46.40]
Trial time as(ms)	906.26[888.3,924.4]	814.48[802.6,826.5]	850.119[829.3,870.9]

#### Gaze Similarity

**Figure 1:**
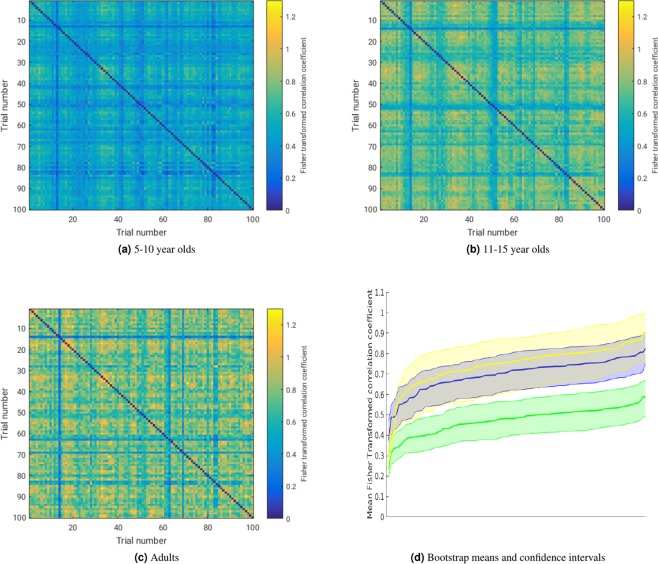
Gaze similarity figures. Panels (a–c) are mean GSMs for each group. Panel (d) is the mean Fisher transformed correlation coefficient, with bootstrap confidence intervals for each trial, sorted by highest value. Data in yellow are from adults, blue from the 11–15 y/o group, and green from the 5–10 y/o group.

In addition to looking at global eye movement characteristics, we investigated the variability in gaze patterns across the trials and age groups through gaze similarity matrices (GSMs). GSMs are based on pairwise correlations between the trials’ smoothed gaze maps. Thus, GSMs reveal the variability or consistency in gaze locations through the experiment (across trials). Figure 1d shows, for each of the 100 trials, the average correlation between its gaze map and the gaze maps generated by the other 99 trials. The trials are sorted according to how consistent their gaze map is compared to all the other trials. The shaded areas represent bootstrap confidence intervals across participants. Figure 1a–c suggests that 5–10 y/os have the least consistency in gaze behaviours across trials, while adults are the most consistent. This is illustrated most clearly by Fig. 1d which shows that 5–10 y/os produce significantly less consistent gaze patterns across trials compared to 11–15 y/os, and adults, who do not differ from each other.

#### Statistical Mapping

Statistical mapping using iMap4^55^ allowed us to spatially isolate the effect of age on gaze pattern. Moreover, we explored how distractors and task difficulty specifically impact on gaze distribution across ages.

**Figure 2:**
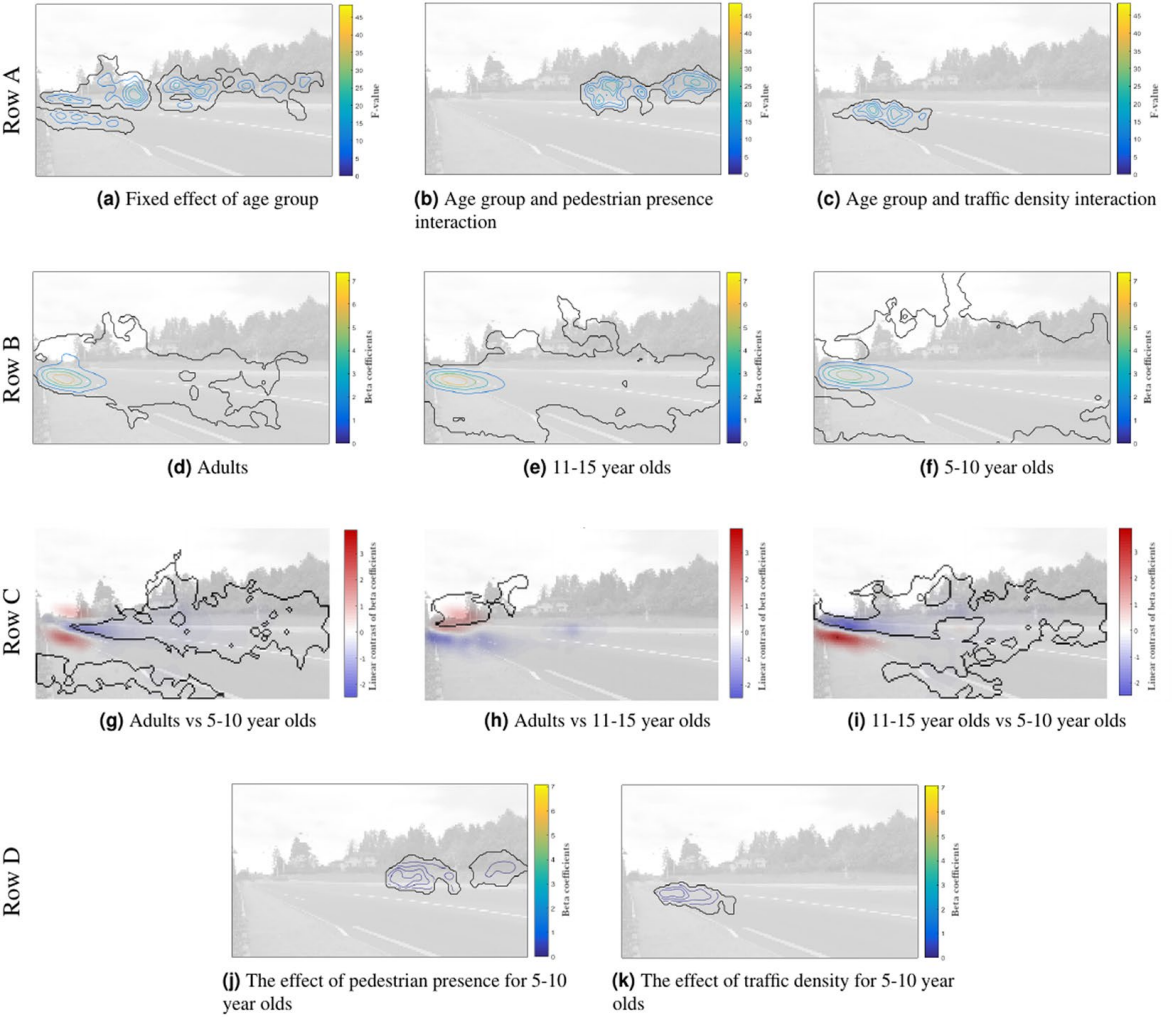
iMap4 analysis. (**A**) Statistical gaze maps with colour coding indicating the F-values. (**B**) Beta maps for each age group. (**C**) Pairwise contrasts between each age group. Warm colours indicate areas where the older age group looked more, compared to the younger age group. (**D**) Effect of pedestrian presence and traffic density for 5–10 y/os.

iMap analysis revealed that age group impacted on the favoured gaze location on the videos. The statistical map for the main effect of age (Fig. 2a) shows significant differences at the beginning of the vehicle’s trajectory and the sidewalks. This age effect can be characterised by representing the differential gaze distributions for each age group. More precisely, older participants maintain their gaze within a smaller area (Fig. 2d–f) – adults gaze mainly at the beginning of the vehicle’s trajectory while 11–15 and 5–10 y/os progressively show a wider gaze distribution covering the sidewalks and a larger proportion of the road (significant areas 2830, 3409 and 4183 pixels for adults, 11–15 y/os and 5–10 y/os respectively). Figure 2g–i illustrates this by representing pairwise contrast between all age groups, depicting statistical differences in gaze distributions across age groups.

The interaction between age group and pedestrian presence (Fig. 2b) reveals a significant area over the sidewalks. The interaction between age group and traffic density (Fig. 2c) shows a significant area on the part of the road corresponding to approaching vehicles. These significant interactions were investigated further via simple effects of pedestrian presence and traffic density for each age group (Fig. 2j,k). The effect of pedestrian presence and traffic density were only significant for 5–10 y/os (Fig. 2j). When a pedestrian was present in the videos, 5–10 y/o children looked more at the sidewalks (Fig. 2k) which was not the case for 11–15 y/os and adults. When the traffic was dense (more than 3 vehicles on screen) 5–10 y/o children looked further down the vehicle’s trajectory (compared to the maximum of their gaze distribution, at the appearing point). Such an effect was not observed for 11–15 y/os and adults.

### Road-crossing decisions

**Figure 3:**
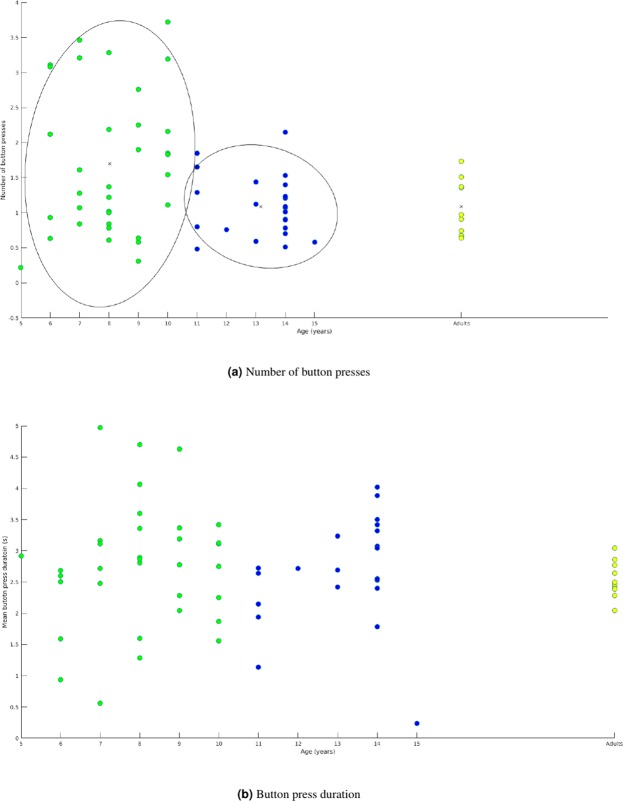
The mean number of crossing decisions (**a**) and mean button press durations (**b**) per trial for individual participants. Each figure is a scatter plot with coloured dots indicating the different groups determined by k-means clustering. The yellow scatter points are the adult group data, blue represent 11–15 y/os, and green are 5–10 y/os. The ellipses highlight the clusters identified by k means (button press number only).

A k-means analysis on the mean number of crossing decisions per participant corroborated differences in performance for children below and above 11 y/o. Indeed, the k-means procedure isolated the following clusters: 5–10 y/os (mean = 8, SD = 1) and 11–15 y/os (mean = 13, SD = 1). The Yuen’s test showed 5–10 y/os made significantly more button presses than 11–15 y/os (t = 10.70, df = 3414, p < 0.05, d = 0.29, see Fig. 3a) and adults (t = 9.86, df = 2410, p < 0.05, d = 0.25). Contrastingly, 11–15 y/os do not differ from adults in the number button presses (t = 1.27,df = 1755,p = 0.20,d = −0.043). The Yuen’s test showed 5–10 y/os pressed for longer than adults (t = 8.42, df = 1650.77, p < 0.05, d = 0.25; see Fig. 3b) and 11–15 y/os (t = 8.25, df = 3361.33, p < 0.05, d = 0.23). 11–15 y/os did not press differently from adults (t = 0.876, df = 1440.98, p = 0.38, d = 0.03).

## Discussion

We recorded eye movements of adults and children while they watched videos of road traffic and were asked to decide when they believed they could cross the road. Eye movement data showed that 5–10 y/os exhibited a much less systematic gaze scanpath than older children or adults. Indeed, older children and adults mainly looked at the beginning of the vehicle’s trajectory. In contrast, younger children showed sparse gaze distributions, covering sidewalks and the vehicle’s trajectory closer to them. All age groups showed disruptions in general oculomotor characteristics depending on the presence of pedestrians in the scene. However, only younger children showed direct gazing at the areas with pedestrian distractors. The traffic density had an effect on younger children’s general oculomotor characteristics, with more fixations in locations closer to the observer. The crossing decision results are consistent with previous literature^56,57^, and confirm a critical age of 10, under which children made more crossing decisions.

The higher number of road crossing decisions for 5–10 y/os compared to adults and older children were associated with gaze pattern biases. The young children’s gaze patterns were characterised by less consistency across trials and more spread across the stimulus space. More specifically, younger children looked significantly more at the sidewalk area than adults and older children when human beings were present in the scene. This suggests that human beings attract the overt attention of younger children but not of older children and adults. Interestingly, human beings in the scene disrupted general oculomotor measures (more fixations, fewer pursuits, smaller proportion of trial as pursuit, and larger proportion of trial as fixation) for all age groups. Hence, it seems that socially relevant stimuli (including faces, body motion, etc.) capture the covert attention of all age groups but that only younger children direct their gaze towards this type of stimuli, which are irrelevant to the crossing task. It is possible that older children and adults are able to inhibit saccades towards irrelevant stimuli, while younger children are lacking the inhibitory control to do so. This scenario is consistent with the findings of psychophysical and neuroscientific studies that children are less able to inhibit automatic saccades, instead directing their overt attention towards task irrelevant stimuli, which is linked in the literature to the ongoing maturation of executive functions due to a protracted maturation of frontal lobes^33,34^.

In all traffic density situations, adults and 11–15 y/os look at the top-left of the road in our paradigm – the appearing point of the cars. We propose that the reason this strategy is used is that this location represents an ideal fixation position for assessing the vehicle’s speed and time to impact as early as possible. Moreover, this gaze location allows the pedestrians to monitor for new vehicles entering the lane, thus to detect gaps, or end of gaps, very early. As the vehicles approach closer to the pedestrians, they could easily be tracked using peripheral vision as their retinal projection gets larger. Children aged between 5 and 10 also appear to be able to use this strategy, as their gaze is also focused on the appearing point of the car. However, in high traffic density situations, children look at the appearing point, as well as further down the road. We suggest that this is because they are following the cars down the road with their gaze, rather than just gazing at the appearing point. This may be due to an inability of 5–10 y/os to disengage their attention from task irrelevant stimuli, once their attention has been drawn by them. This hypothesis is consistent with studies showing that individuals in general are drawn to stimuli before disengaging to focus their attention on the target stimuli^58,59^. While pursuing the vehicles, the observers’ attention is focused on the vehicle moving down the road and is not be able to attend to other vehicles entering the road. This scenario is in line with studies showing that participants are not able to allocate much attention to objects in the periphery while pursuing a target^60,61^. Without accurate information about vehicle position children would not be able to make informed crossing decisions which could lead them to cross unsafely.

Overall, our results show systematic links between eye movement patterns and road crossing decisions across development. We propose that gaze locations have a direct impact on crossing decisions. Children orient their overt attention towards human distractors more than 11–15 y/os and adults. This tendency would impair their ability to attend to the vehicles, thus making accurate judgements about a vehicle’s distance more difficult.

Our study provides new important insights in children’s deficits in attentional control in realistic situations, particularly their vulnerability as pedestrians. Our findings are consistent with recent studies that show a very similar pattern of development. In real life situations, Connelly and colleagues^56^ demonstrated that children below 11 years of age do not make safe decisions. Simpson, Johnston, and Richardson^62^ reach similar conclusions using a VR head mounted display. Some recent studies used immersive VR environments allowing for realistic pedestrian^63^ or cyclist actions^64,65^, and unveiled the developmental trajectory of the fine-tuning between perception, decision, and action.

This study isolated, for the range of situations tested, the critical age from which children’s attentional control is at adult level in a road crossing task. Children below 11 years of age show differences in their visual explorations, characterised by a more spread gaze distribution, more overt attention to stimuli irrelevant to the task, and more gazing following the vehicles closer to the participant. This specific oculomotor pattern was associated with riskier crossing decisions in shorter traffic gaps compared to older children and adults. Our findings suggest that below 11 years of age, children do not employ attentional control to a level required for safe crossing decisions. Thus, training and education programs might specifically target these vulnerable children and their caregivers. It is also important to note that our task incorporated only one traffic direction. Thus, the critical age might occur even later in more complex and taxing situations incorporating two traffic directions.

This work helps us to better understand general deficits in children’s attentional control in real world situations, and in particular their vulnerability as pedestrians. In future studies, these initial findings will be supplemented by ongoing research investigating questions such as the visual exploration in 3D, fine-grained analyses of time to impact and moment by moment crossing decisions, the mechanisms of attentional disengagement, the neural correlates of visuo-attentional processes for children as pedestrians, and large fields of view with two traffic directions involving eye and head movement coordination.

## Methods

All data is publicly available via the Open Science Framework through this link: 10.17605/OSF.IO/B3YPC.

### Participants

67 participants were recruited: 57 aged between five and 15, and 10 adult controls aged between 20 and 40 (mean = 24, SD = 3). All children were recruited in schools in the Fribourg canton, Switzerland. Adults were recruited from the University of Fribourg. All participants had normal or corrected to normal vision. The study was approved by the Department of Psychology ethics committee at the University of Fribourg. Informed consent was obtained from the schools, parents, children, and adult controls prior to taking part in the study. This study was performed in accordance with all appropriate institutional and international guidelines and regulations, in line with the principles of the Declaration of Helsinki.

### Apparatus

During the experiment participants’ eye movements were recorded at a sampling rate of 1000 Hz with the SR-Research EyeLink 1000 (with a chin and forehead rest), which has an average gaze position error of 0.25°, a spatial resolution of 0.01°, and a linear output range over the range of the monitor used. Only the dominant eye was tracked. Stimuli were presented on an HP monitor with a screen resolution of 1920 by 1080 pixels, a width of 521 mm and a height of 293 mm, a horizontal viewing angle of 46.9° and a vertical viewing angle of 27.4° at a distance of 600 mm. The experiment was coded in Matlab^66^ using Psychophysics (PTB-3) and EyeLink Toolbox extensions^67,68^. Calibrations for eye fixations were conducted at the beginning of the experiment using a nine-point fixation procedure as implemented in the EyeLink API (see EyeLink Manual) and using Matlab software. Calibrations were then validated with EyeLink software and repeated until the optimal calibration criterion was reached.

### Experimental Design

**Figure 4:**
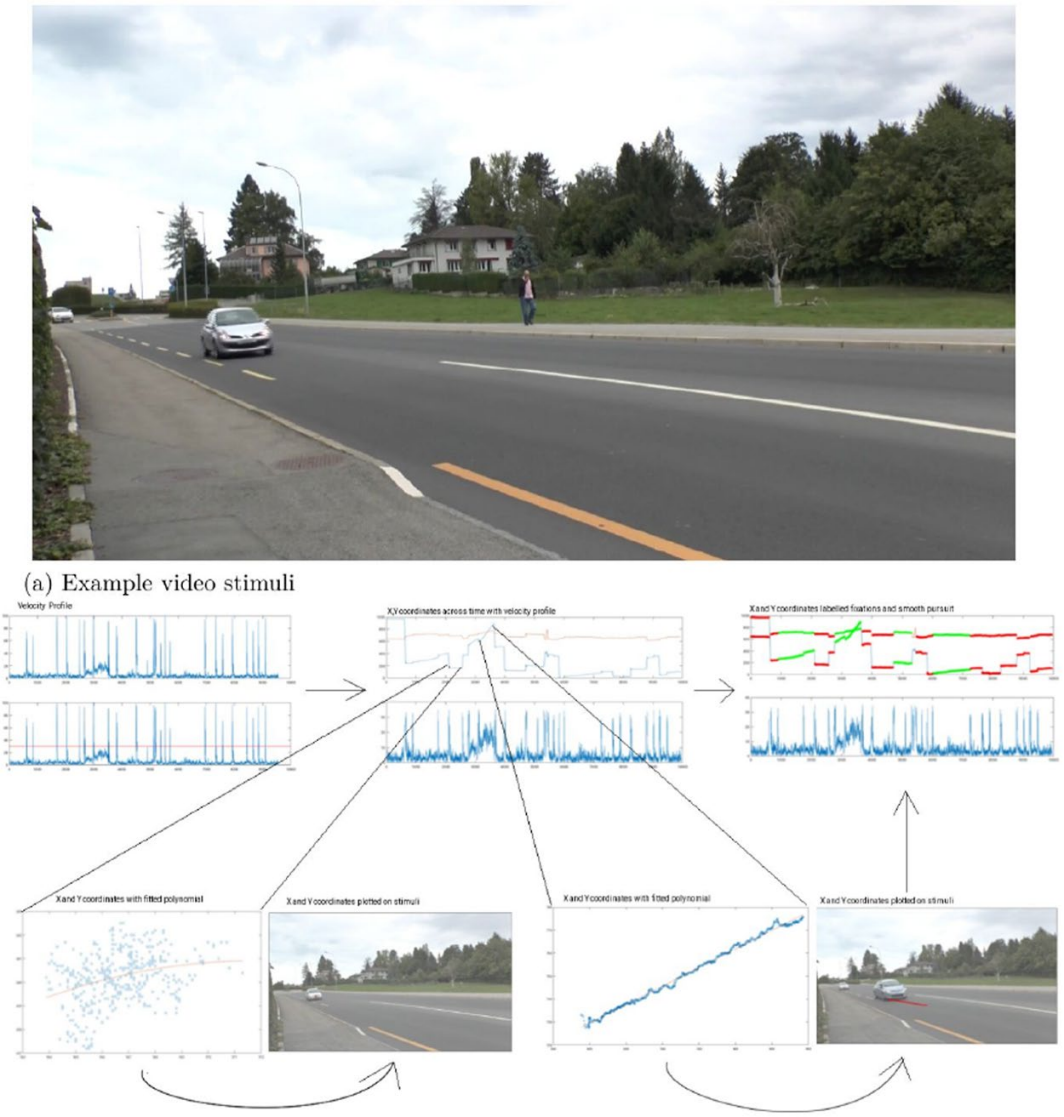
Example video stimuli and illustration of eye parser algorithm (**a**) A screenshot taken from the a video clip shown during a single trial of the experiment. The videos are filmed at an angle, so the participants can see the approach of vehicles only from one side of the road. (**b**) Top left – velocity threshold to extract saccades (bottom panel). Velocity of eye movement samples (top panel). Top centre – plotting X and Y coordinates of eye movement samples across whole trial (top panel). Bottom left and right – extraction of segments of eye movement samples maintaining a velocity of 30 deg/s for at least 100 ms with a polynomial fitted to the segments. Beside these are X and Y coordinates of the segments plotted on matching frames of the experiment stimuli. Top right – completed labelling of eye movements as fixations (red lines), smooth pursuits (green lines), and saccades (blue lines) for a whole trial.

At the beginning of the experiment participants were informed that they would be presented with a series of videos of road crossing situations on screen and that they would have to indicate by pressing the spacebar on a keyboard when they could cross the road and hold the button pressed for as long as they thought it was safe to cross. Participants were instructed to focus on approaching vehicles on the side of the road closest to them (see Fig. 4a for a capture of the scene). Vehicles travelled at an average velocity of 50 km/h. Each trial started with the presentation of a central fixation cross. Once the participants had fixated on the cross a blank screen was presented for 500 ms and then the video clip for the trial was presented (see Fig. 4a). Each trial was followed by another blank screen for 500 ms and the next trial started with the central cross. 100 trials were presented to the participants each with a different video clip, each lasting 10 seconds. All video clips were filmed at a real road crossing in Fribourg with a variety of traffic densities, with or without pedestrians and cyclists (distractors). Number of presses for each trial were collected and analysed for the purpose of the present experiment.

### Statistical Analyses

All statistical analyses and figures were performed and created using Matlab 2016a^66^ and R^69^ with RStudio^70^.

The literature suggests that crossing decisions are different below and above 10 years old. We corroborated this critical age, in a data-driven way to avoid confirmation bias, using a k-means clustering on the mean number of crossing decisions per participant (Fig. 3a). We used the Matlab k-means function, based on the k-mean++ algorithm, and ran 1000 iterations to verify that the centroids were grouping consistently. The k-means procedure isolated the following clusters: 5–10 y/os (mean = 8, SD = 1) and 11–15 y/os (mean = 13, SD = 1). The number of and duration of button presses were analysed using a Yuen’s test with 20% trimmed means in R using the WRS2 package^71^. Eye movements were parsed into fixations, saccades, and smooth pursuits using a custom algorithm. Saccades were extracted using the same parameters as the EyeLink software (a velocity threshold = 50 deg/s). If the majority of samples in the trial were above this threshold then the trial was removed and if more than 50% of the trials were removed then the participant was excluded. In total, 31 trials were removed, and five participants were excluded for noisy recording. Potential smooth pursuit segments were first isolated as segments for which velocity was maintained below or equal to 30 deg/s for a minimum of 100 ms. From this initial extraction, smooth pursuit segments were identified using a dispersion threshold, based on the following algorithm. A polynomial was fitted to the X and Y coordinates of the gaze samples in each smooth eye movement segment, after having removed outliers using the Corr v2 toolbox^72^. The root-mean square error of the polynomial fit was then calculated and divided by the exponential of the arc length (calculated using the arclength toolbox^73^) of the polynomial. A threshold was set at 1 × 10^−9^ and samples below that threshold were considered as smooth pursuit, while samples above were considered as part of a fixation. This algorithm is summarized in Fig. 4b and the following equation:

$${P}_{RMSE}/\exp (A)$$

*P*_*RMSE*_ is the root mean square error of the polynomial line, *A* is the arc length of the polynomial line. For each video clip the presence of a human distractor was encoded in a dichotomous way (1 for one or more human distractors present in the trial, 0 for no human distractors in the trial). The number of vehicles on each trial (traffic density) was determined using Matlab’s computer vision toolbox^74^. This toolbox uses a background subtraction algorithm involving Gaussian mixture models to detect the foreground of each frame of the video. This is followed by a blob analysis to detect and count moving objects – the vehicles in the trial videos (for an example see^75^).

Oculomotor characteristics were analysed using shift functions that were run in R using code from^76^. The oculomotor characteristics included fixation, pursuit and saccade durations, number, and proportion of trial time. The shift functions were produced for the nine oculomotor characteristics according to age group, presence of human distractors, and traffic density. High and low traffic density categories were produced using a kernel density plot of the number of cars on each trial (Fig. S1 in the Supplemental Material). The centre of the distribution of car traffic density was found to be three cars present in the trial. Gaze samples were further analysed using gaze similarity matrices (GSMs). GSMs were computed by creating, for each participant and each trial, smoothed (1° of visual angle) Z-scored maps of the gaze positions as in^55^. The Fisher transformed correlations of the gaze map on a single trial with the gaze maps for all other trials were calculated for each participant individually (Fig. 1a–c). Finally, the mean similarity between the gaze map on a single trial and all the other maps were computed for each participant, leading to 100 values per participant that were used to compute the age group with bootstrapped confidence intervals (Fig. 1d).

Statistical maps were calculated with the iMap toolbox, version 4^55^. iMap computes pixel-wise linear mixed models (LMMs) across participants and trials on each z-score map. iMap uses a universal bootstrap clustering test to resolve biases in parameter estimation and problems arising from multiple comparisons^77,78^. The LMM included pedestrian presence, traffic density, and age group as fixed effects. The model also included random intercepts for subject and video stimuli. Initially the model included random slopes of age group, pedestrian presence, and traffic density for each random intercept; however, this initial model did not converge so all random slopes were removed.

## Supplementary information

Supplementary Materials

LaTeX Supplementary File

LaTeX Supplementary File

LaTeX Supplementary File

LaTeX Supplementary File

LaTeX Supplementary File

LaTeX Supplementary File

LaTeX Supplementary File

LaTeX Supplementary File

LaTeX Supplementary File

LaTeX Supplementary File

LaTeX Supplementary File

LaTeX Supplementary File

LaTeX Supplementary File

LaTeX Supplementary File

LaTeX Supplementary File

LaTeX Supplementary File

LaTeX Supplementary File

LaTeX Supplementary File

LaTeX Supplementary File

## References

[1] Ludwig CJ, Davies JR, Eckstein MP (2014). Foveal analysis and peripheral selection during active visual sampling. Proceedings of the National Academy of Sciences.

[2] Itti L, Koch C (2001). Computational modelling of visual attention. Nat. Rev. Neurosci..

[3] Borji A, Sihite DN, Itti L (2013). What stands out in a scene? a study of human explicit saliency judgment. Vision Res..

[4] Bruce, N. & Tsotsos, J. Saliency based on information maximization. In *Advances in neural information processing systems*, **18**, 155–162 (NIPS, 2006).

[5] Bruce ND, Tsotsos JK (2009). Saliency, attention, and visual search: An information theoretic approach. Journal of Vision.

[6] Marat S (2009). Modelling spatio-temporal saliency to predict gaze direction for short videos. International Journal of Computer Vision.

[7] Henderson JM, Weeks PA, Hollingworth A (1999). The effects of semantic consistency on eye movements during complex scene viewing. Journal of Experimental Psychology: Human Perception and Performance.

[8] Loftus GR, Mackworth NH (1978). Cognitive determinants of fixation location during picture viewing. Journal of Experimental Psychology: Human Perception and Performance.

[9] Palmer SE (1975). The effects of contextual scenes on the identification of objects. Memory & Cognition.

[10] Tatler BW, Hayhoe MM, Land MF, Ballard DH (2011). Eye guidance in natural vision: Reinterpreting salience. Journal of Vision.

[11] Wolfe JM, Võ ML-H, Evans KK, Greene MR (2011). Visual search in scenes involves selective and nonselective pathways. Trends in Cognitive Sciences.

[12] Eckstein MP, Drescher BA, Shimozaki SS (2006). Attentional cues in real scenes, saccadic targeting, and bayesian priors. Psychological Science.

[13] Henderson JM, Chanceaux M, Smith TJ (2009). The influence of clutter on real-world scene search: Evidence from search efficiency and eye movements. Journal of Vision.

[14] Henderson JM, Malcolm GL, Schandl C (2009). Searching in the dark: Cognitive relevance drives attention in real-world scenes. Psychonomic Bulletin & Review.

[15] Malcolm GL, Henderson JM (2010). Combining top-down processes to guide eye movements during real-world scene search. Journal of Vision.

[16] Võ ML-H, Henderson JM (2010). The time course of initial scene processing for eye movement guidance in natural scene search. Journal of Vision.

[17] Hwang AD, Wang H-C, Pomplun M (2011). Semantic guidance of eye movements in real-world scenes. Vision Res..

[18] Castelhano MS, Heaven C (2011). Scene context influences without scene gist: Eye movements guided by spatial associations in visual search. Psychonomic Bulletin & Review.

[19] Castelhano MS, Henderson JM (2007). Initial scene representations facilitate eye movement guidance in visual search. Journal of Experimental Psychology: Human Perception and Performance.

[20] Võ ML-H, Wolfe JM (2012). When does repeated search in scenes involve memory? looking at versus looking for objects in scenes. Journal of Experimental Psychology: Human Perception and Performance.

[21] Castelhano MS, Pereira EJ (2018). The influence of scene context on parafoveal processing of objects. Quarterly Journal of Experimental Psychology.

[22] Pereira EJ, Castelhano MS (2014). Peripheral guidance in scenes: The interaction of scene context and object content. Journal of Experimental Psychology: Human Perception and Performance.

[23] Hayes TR, Henderson JM (2017). Scan patterns during real-world scene viewing predict individual differences in cognitive capacity. Journal of Vision.

[24] Henderson JM (2017). Gaze control as prediction. Trends in Cognitive Sciences.

[25] Friston K, Adams R, Perrinet L, Breakspear M (2012). Perceptions as hypotheses: saccades as experiments. Frontiers in Psychology.

[26] Booth JR (2003). Neural development of selective attention and response inhibition. NeuroImage.

[27] Bunge SA, Dudukovic NM, Thomason ME, Vaidya CJ, Gabrieli JD (2002). Immature frontal lobe contributions to cognitive control in children: evidence from fmri. Neuron.

[28] Durston S (2002). A neural basis for the development of inhibitory control. Developmental Science.

[29] Kastner S, Ungerleider LG (2000). Mechanisms of visual attention in the human cortex. Annual Review of Neuroscience.

[30] Hwang K, Velanova K, Luna B (2010). Strengthening of top-down frontal cognitive control networks underlying the development of inhibitory control: a functional magnetic resonance imaging effective connectivity study. Journal of Neuroscience.

[31] Konrad K (2005). Development of attentional networks: an fmri study with children and adults. NeuroImage.

[32] Colombo J (2001). The development of visual attention in infancy. Annual Review of Psychology.

[33] Munoz DP, Everling S (2004). Look away: the anti-saccade task and the voluntary control of eye movement. Nature Reviews. Neuroscience.

[34] Paus T (1989). The development of sustained attention in children might be related to the maturation of frontal cortical functions. Acta Neurobiologiae Experimentalis.

[35] Fukushima J, Hatta T, Fukushima K (2000). Development of voluntary control of saccadic eye movements: I. age-related changes in normal children. Brain and Development.

[36] Klein C, Foerster F (2001). Development of prosaccade and antisaccade task performance in participants aged 6 to 26 years. Psychophysiology.

[37] Klein C, Fischer B, Hartnegg K, Heiss W, Roth M (2000). Optomotor and neuropsychological performance in old age. Experimental Brain Research.

[38] Munoz DP, Broughton JR, Goldring JE, Armstrong IT (1998). Age-related performance of human subjects on saccadic eye movement tasks. Experimental Brain Research.

[39] Leclercq V, Siéroff E (2013). Development of endogenous orienting of attention in school-age children. Child Neuropsychology.

[40] Açık A, Sarwary A, Schultze-Kraft R, Onat S, König P (2010). Developmental changes in natural viewing behavior: bottom-up and top-down differences between children, young adults and older adults. Frontiers in Psychology.

[41] Kuhn G, Teszka R (2017). Don’t get misdirected! differences in overt and covert attentional inhibition between children and adults. the Quarterly Journal of Experimental Psychology.

[42] World Health Organization. *Pedestrian safety: a road safety manual for decision-makers and practitioners*. (2013).

[43] World Health Organization. *Ten strategies for keeping children safe on the road* (2015).

[44] Pitcairn T, Edlmann T (2000). Individual differences in road crossing ability in young children and adults. British Journal of Psychology.

[45] Plumert JM, Kearney JK, Cremer JF (2004). Children’s perception of gap affordances: Bicycling across traffic-filled intersections in an immersive virtual environment. Child Development.

[46] te Velde AF, van der Kamp J, Barela JA, Savelsbergh GJ (2005). Visual timing and adaptive behavior in a road-crossing simulation study. Accid. Anal. & Prev..

[47] Schwebel DC, Davis AL, O’Neal EE (2012). Child pedestrian injury: A review of behavioral risks and preventive strategies. American Journal of Lifestyle Medicine.

[48] Zeedyk MS, Wallace L, Spry L (2002). Stop, look, listen, and think?: What young children really do when crossing the road. Accid. Anal. & Prev..

[49] Zeedyk MS, Kelly L (2003). Behavioural observations of adult–child pairs at pedestrian crossings. Accid. Anal. & Prev..

[50] Morrongiello BA, Corbett M, Milanovic M, Beer J (2015). Using a virtual environment to examine how children cross streets: Advancing our understanding of how injury risk arises. Journal of Pediatric Psychology.

[51] Barton BK, Schwebel DC (2007). The roles of age, gender, inhibitory control, and parental supervision in children’s pedestrian safety. Journal of Pediatric Psychology.

[52] Whitebread D, Neilson K (2000). The contribution of visual search strategies to the development of pedestrian skills by 4-11 year-old children. British Journal of Educational Psychology.

[53] Tapiro, H., Meir, A., Parmet, Y. & Oron-Gilad, T. Visual search strategies of child-pedestrians in road crossing tasks. In de Waard, D. *et al*. (eds) *Proceedings of the Human Factors and Ergonomics Society* (Perception, 2014).

[54] Caldara R, Miellet S (2011). imap: a novel method for statistical fixation mapping of eye movement data. Behavior Research Methods.

[55] Lao J, Miellet S, Pernet CR, Sokhn N, Caldara R (2017). imap4: An open source toolbox for the statistical fixation mapping of eye movement data with linear mixed modeling. Behavior Research Methods.

[56] Connelly ML, Conaglen HM, Parsonson BS, Isler RB (1998). Child pedestrians’ crossing gap thresholds. Accid. Anal. & Prev..

[57] Lee DN, Young DS, McLaughlin CM (1984). A roadside simulation of road crossing for children. Ergonomics.

[58] Kim M-S, Cave KR (1999). Top-down and bottom-up attentional control: On the nature of interference from a salient distractor. Atten. Percept. Psychophys..

[59] Theeuwes, J., Atchley, P. & Kramer, A. F. On the time course of top-down and bottom-up control of visual attention. In Monsell, S. & Driver, J. (eds) *Control of cognitive processes: Attention and performance XVIII*, 105–124 (MIT Press, Cambridge,MA, 2000).

[60] Hutton S, Tegally D (2005). The effects of dividing attention on smooth pursuit eye tracking. Experimental Brain Research.

[61] Lovejoy LP, Fowler GA, Krauzlis RJ (2009). Spatial allocation of attention during smooth pursuit eye movements. Vision Res..

[62] Simpson G, Johnston L, Richardson M (2003). An investigation of road crossing in a virtual environment. Accident Anal. & Prevention.

[63] O’Neal EE (2017). Changes in perception–action tuning over long time scales: How children and adults perceive and act on dynamic affordances when crossing roads. Journal of Experimental Psychology: Human Perception and Performance.

[64] Grechkin TY, Chihak BJ, Cremer JF, Kearney JK, Plumert JM (2013). Perceiving and acting on complex affordances: How children and adults bicycle across two lanes of opposing traffic. Journal of Experimental Psychology: Human Perception and Performance.

[65] Chihak BJ (2010). Synchronizing self and object movement: How child and adult cyclists intercept moving gaps in a virtual environment. Journal of Experimental Psychology: Human Perception and Performance.

[66] MATLAB. *version 7.10.0 (R2016a)* (The MathWorks Inc., Natick, Massachusetts, 2016).

[67] Brainard DH (1997). The psychophysics toolbox. Spatial vision.

[68] Cornelissen FW, Peters EM, Palmer J (2002). The eyelink toolbox: eye tracking with matlab and the psychophysics toolbox. Behavior Research Methods, Instruments, Computers.

[69] R Core Team. *R: A Language and Environment for Statistical Computing*. R Foundation for Statistical Computing, Vienna, Austria, https://www.R-project.org (2016).

[70] RStudio Team. *RStudio: Integrated Development Environment for R*. RStudio, Inc., Boston, MA, http://www.rstudio.com/ (2016).

[71] Mair, P. & Wilcox, R. *WRS2: Wilcox robust estimation and testin*g, http://CRAN.R-project.org/package=WRS2 0.10-0 (2018).

[72] Pernet CR, Wilcox RR, Rousselet GA (2013). Robust correlation analyses: false positive and power validation using a new open source matlab toolbox. Frontiers in Psychology.

[73] D’Errico, J. *arclength* (MATLAB Central File Exchange, https://uk.mathworks.com/matlabcentral/fileexchange/34871-arclength.1.0 (2010)

[74] MATLAB. *MATLAB Computer Vision System Toolbox* (The MathWorks Inc., Natick, Massachusetts, 2016a).

[75] Kingdom, M. U. Detecting cars using gaussian mixture models- matlab and simulink example, https://uk.mathworks.com/help/vision/examples/detecting-cars-using-gaussian-mixture-models.htmlresponsiveoffcanvas.1.0 (2017).

[76] Rousselet GA, Pernet CR, Wilcox RR (2017). Beyond differences in means: robust graphical methods to compare two groups in neuroscience. European Journal of Neuroscience.

[77] Pernet, C. R., Chauveau, N., Gaspar, C. & Rousselet, G. A. Limo eeg: A toolbox for hierarchical linear modeling of electroencephalographic data. *Intell. Neuroscience* **2011**, 10.1155/2011/831409 (2011). 10.1155/2011/831409 PMC3049326 21403915

[78] Pernet CR, Latinus M, Nichols TE, Rousselet GA (2015). Cluster-based computational methods for mass univariate analyses of event-related brain potentials/fields: A simulation study. Journal of Neuroscience Methods.

