# Supplementary material for: Developing attentional control in naturalistic dynamic road crossing situations
Source: Sci Rep. 2019 Mar 12;9:4176. doi: 10.1038/s41598-019-39737-7 (PMC6414534; doi:10.1038/s41598-019-39737-7)
Supplement: Supplementary file 1 — Supplementary Materials [file 41598_2019_39737_MOESM1_ESM.pdf]

Developing attentional control in naturalistic dynamic road crossing situations

Victoria I. Nicholls[1], Geraldine Jean-Charles[2], Junpeng Lao[2], Peter de Lissa[2], Roberto Caldara[2], and Sebastien Miellet[3*]

[1]Bournemouth University, Faculty of Science & Technology, Bournemouth, BH12 1PH, United Kingdom
[2]University of Fribourg, Department of Psychology, iBM lab, Fribourg, 1700, Switzerland
[3]University of Wollongong, School of Psychology, ActiveVision lab, Wollongong, 2522, Australia
[*]smiellet@uow.edu.au

Supplementary Materials

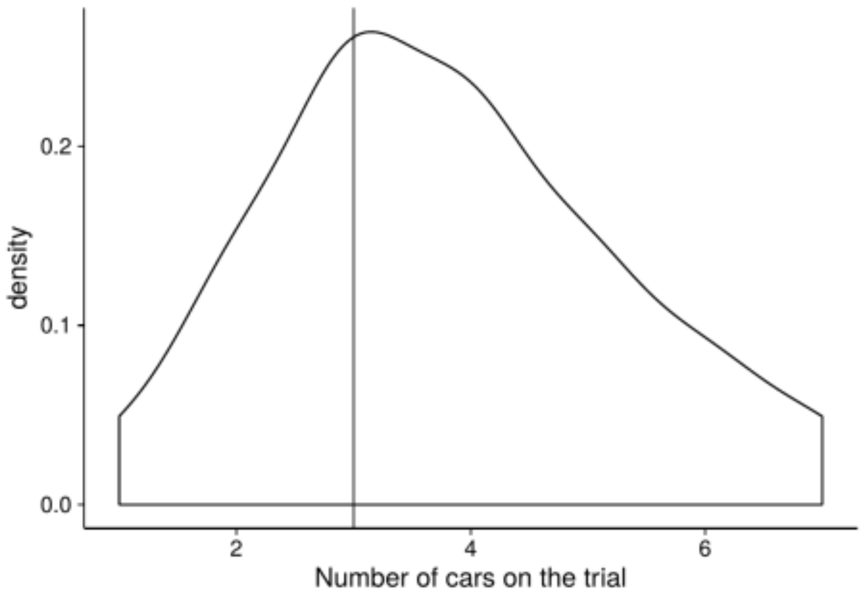

Figure S1. Density of trials with each number of cars present. The kernel density plot shows the density of trials with each number of cars present, ranging from 1 to 7 cars present in a trial. The figure was used to determine 'high traffic' and 'low traffic' density trials, to assess the influence of traffic density on the low level eye movement parameters (the number of fixations, durations of pursuits, etc). The peak of the kernel density appears at 3 cars present on the trial. From this we labelled 3 cars in the trial or fewer to be low traffic density and more than 3 cars in the trial to be high traffic density.

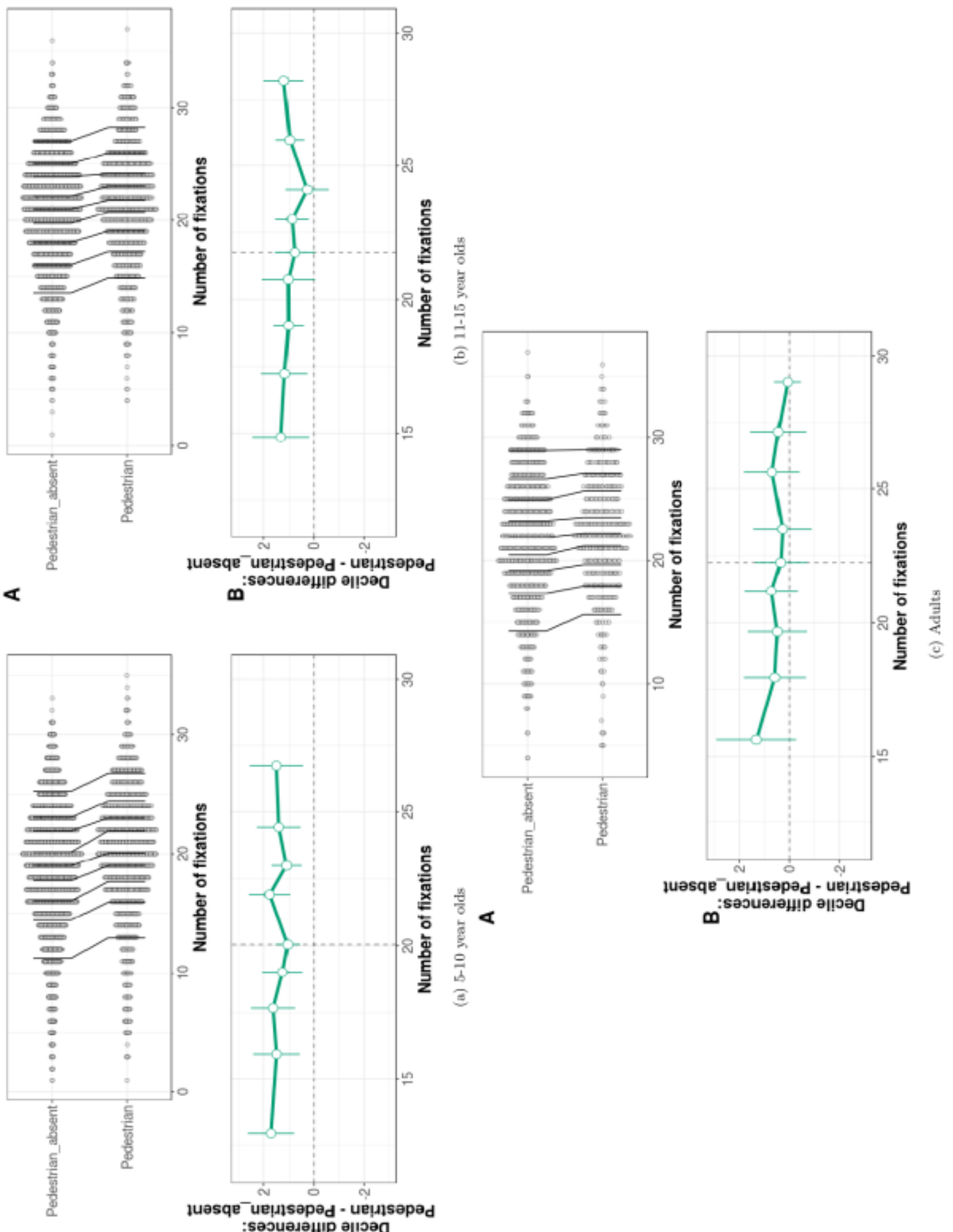

(a) 5-10 year olds

(b) 11-15 year olds

(c) Adults

Figure S2. Shift functions for number of fixations with pedestrian presence Panel A for each figure indicates scatter plots for the number of fixations in trials – the points are jittered by their local density. The black lines indicate the estimated deciles for each group, as well as the difference between them. Panel B for each figure shows the shift function for the two distributions. The shift functions show the difference between the number of each gaze sample type for each decile – white discs – on trials where pedestrians are present and trials where pedestrians are not present. The error bars indicate 95% confidence intervals. A significant difference is present when the error bars do not include zero. (a) shows the difference in the number of fixations for 5-10y/os, (b) for 11-15y/os, and (c) for adults on trials where pedestrians are present and trials where they are not present. For more details on the shift functions see:[77].

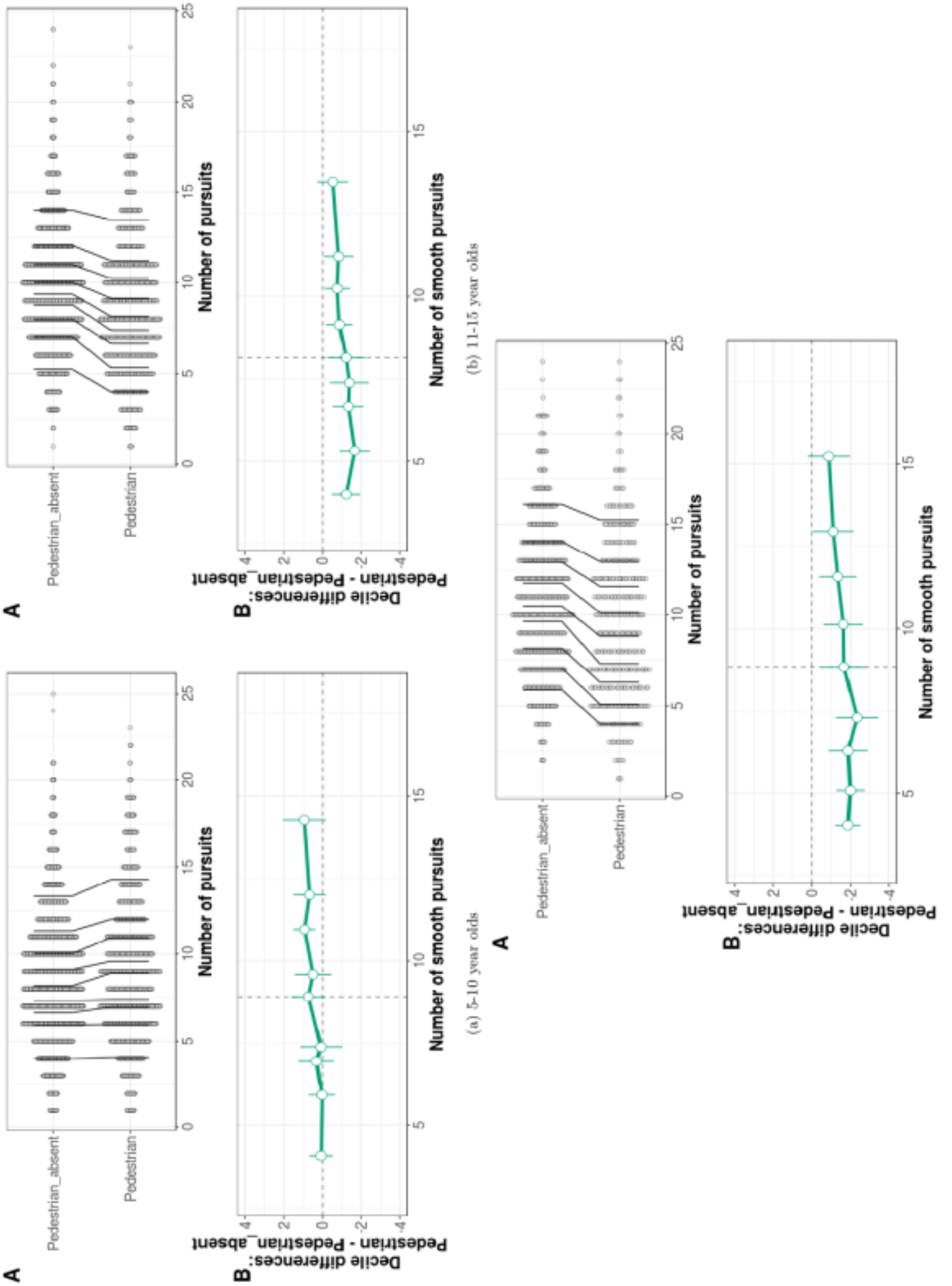

Figure S3. **Shift functions for the number of smooth pursuits with pedestrian presence** (a) shows the difference in the number of smooth pursuits for 5-10y/os, (b) for 11-15y/os, and (c) for adults on trials where pedestrians are present and trials where pedestrians are not present.

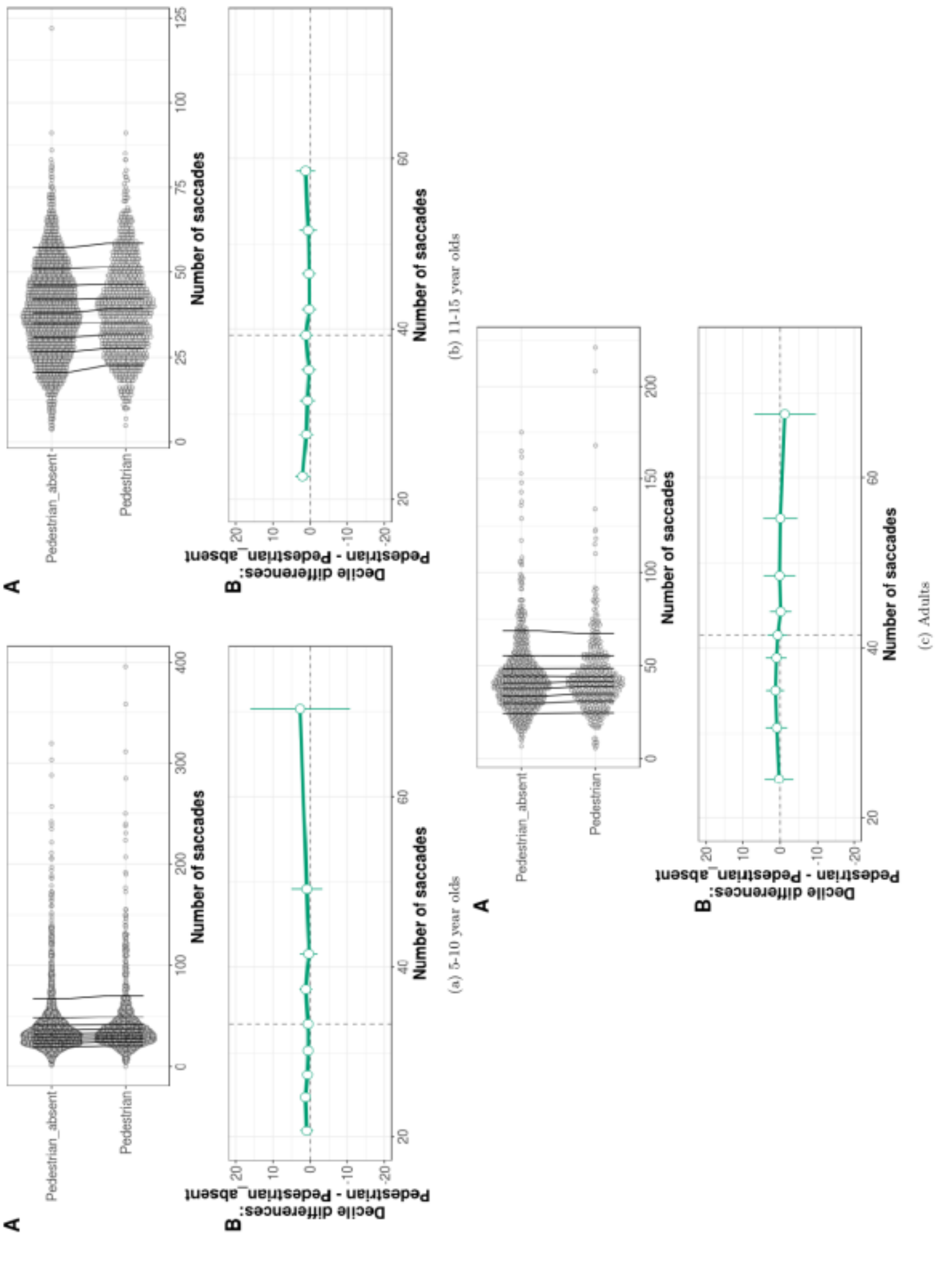

Figure S4. Shift functions for the number of saccades with pedestrian presence. (a) shows the difference in the number of saccades for 5-10y/os, (b) for 11-15y/os, and (c) for adults on trials where pedestrians are present and trials where pedestrians are not present.

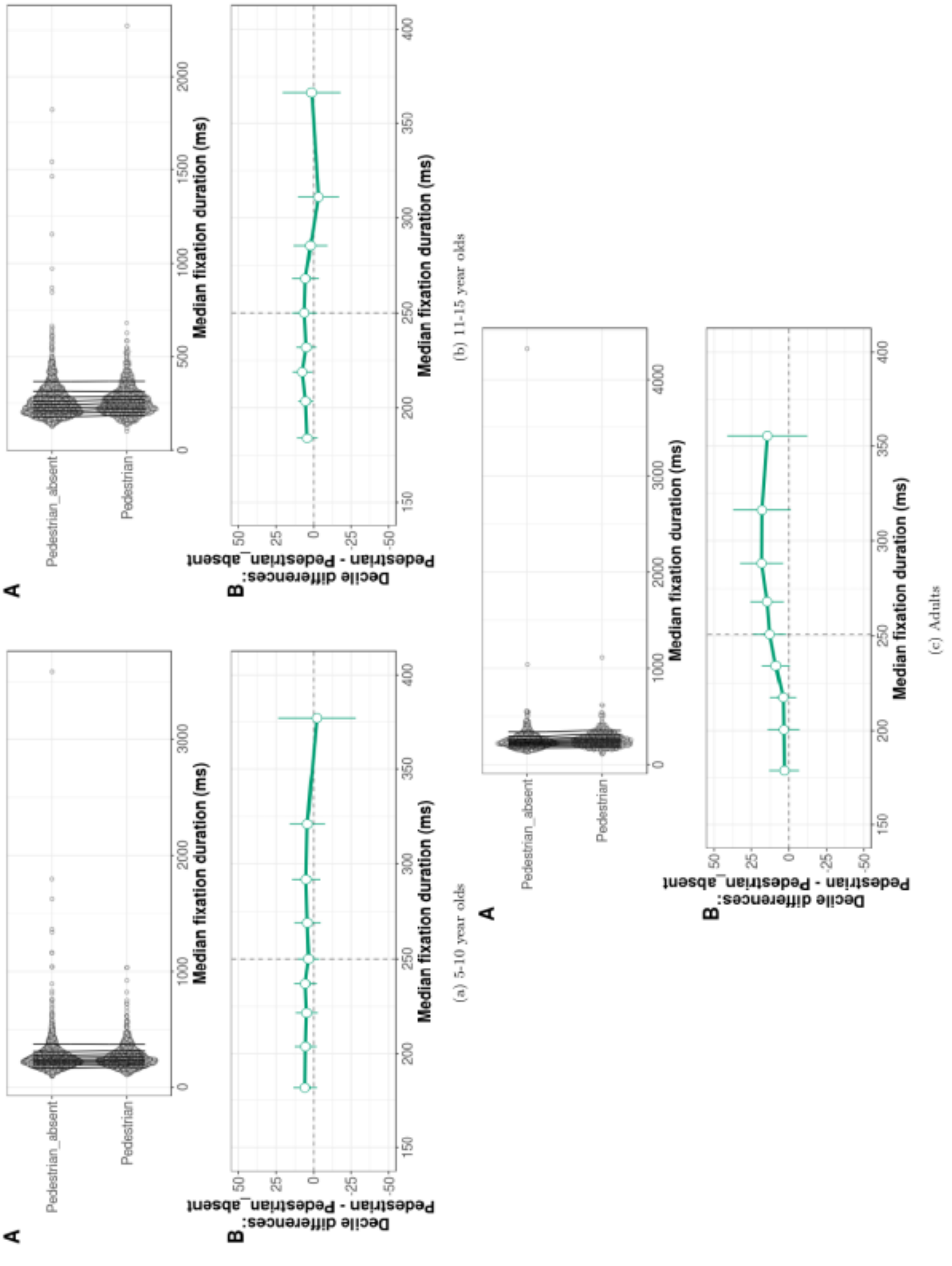

Figure S5. Shift functions for the median duration of fixations with pedestrian presence. (a) shows the difference in the median duration of fixations for 5-10y/os, (b) for 11-15y/os, and (c) for adults on trials where pedestrians are present and trials where pedestrians are not present.

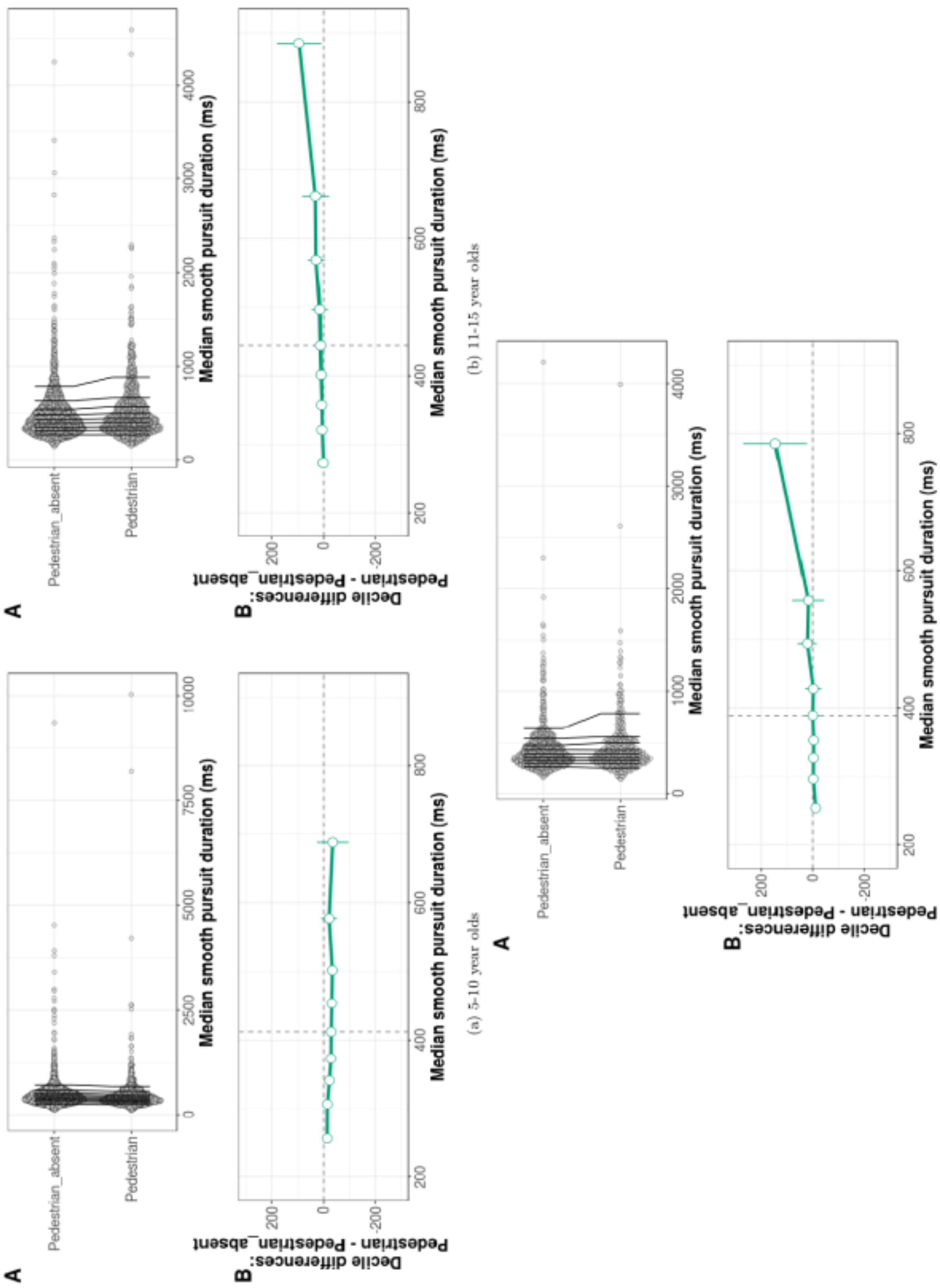

Figure S6. Shift functions for the median duration of smooth pursuits with pedestrian presence. (a) shows the difference in the median duration of smooth pursuits for 5-10y/os, (b) for 11-15y/os, and (c) for adults on trials where pedestrians are present and trials where pedestrians are not present.

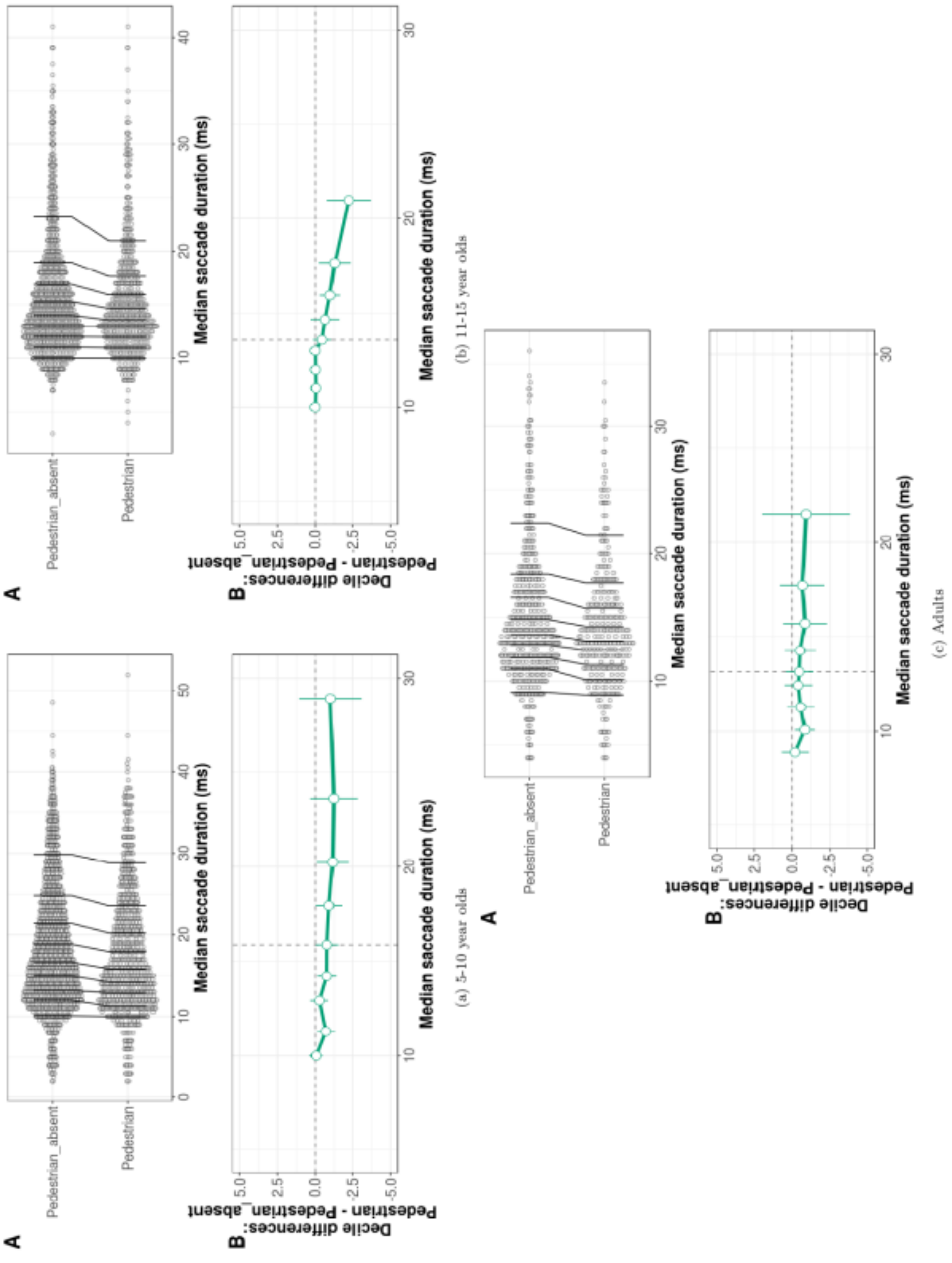

Figure S7. Shift functions for the median duration of saccades with pedestrian presence. (a) shows the difference in the median duration of saccades for 5-10y/os, (b) for 11-15y/os, and (c) for adults on trials where pedestrians are present and trials where pedestrians are not present.

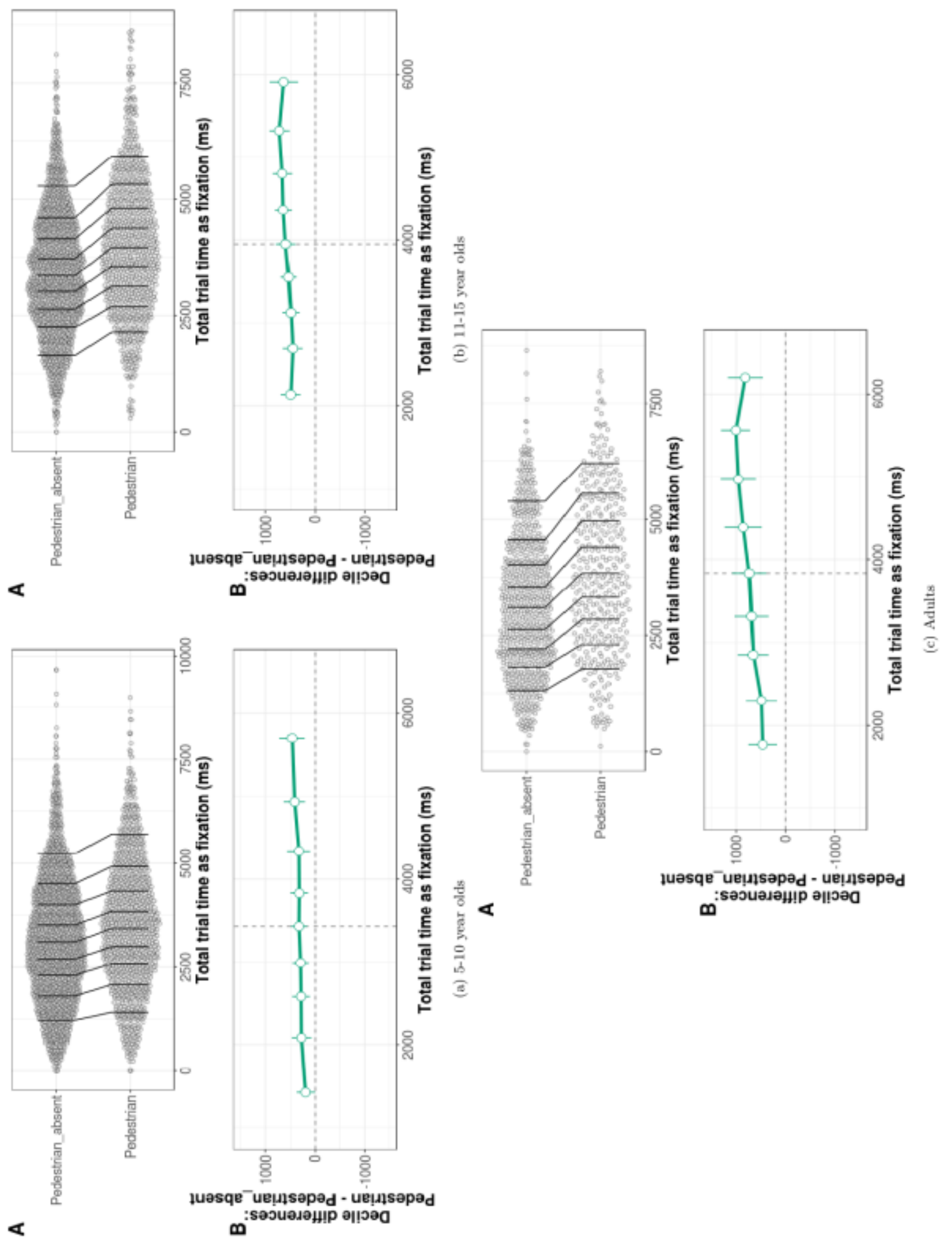

Figure S8. Shift functions for the proportion of trial time as fixation with pedestrian presence (a) shows the difference in the proportion of trial time as fixation for 5-10y/os, (b) for 11-15y/os, and (c) for adults on trials where pedestrians are present and trials where pedestrians are not present.

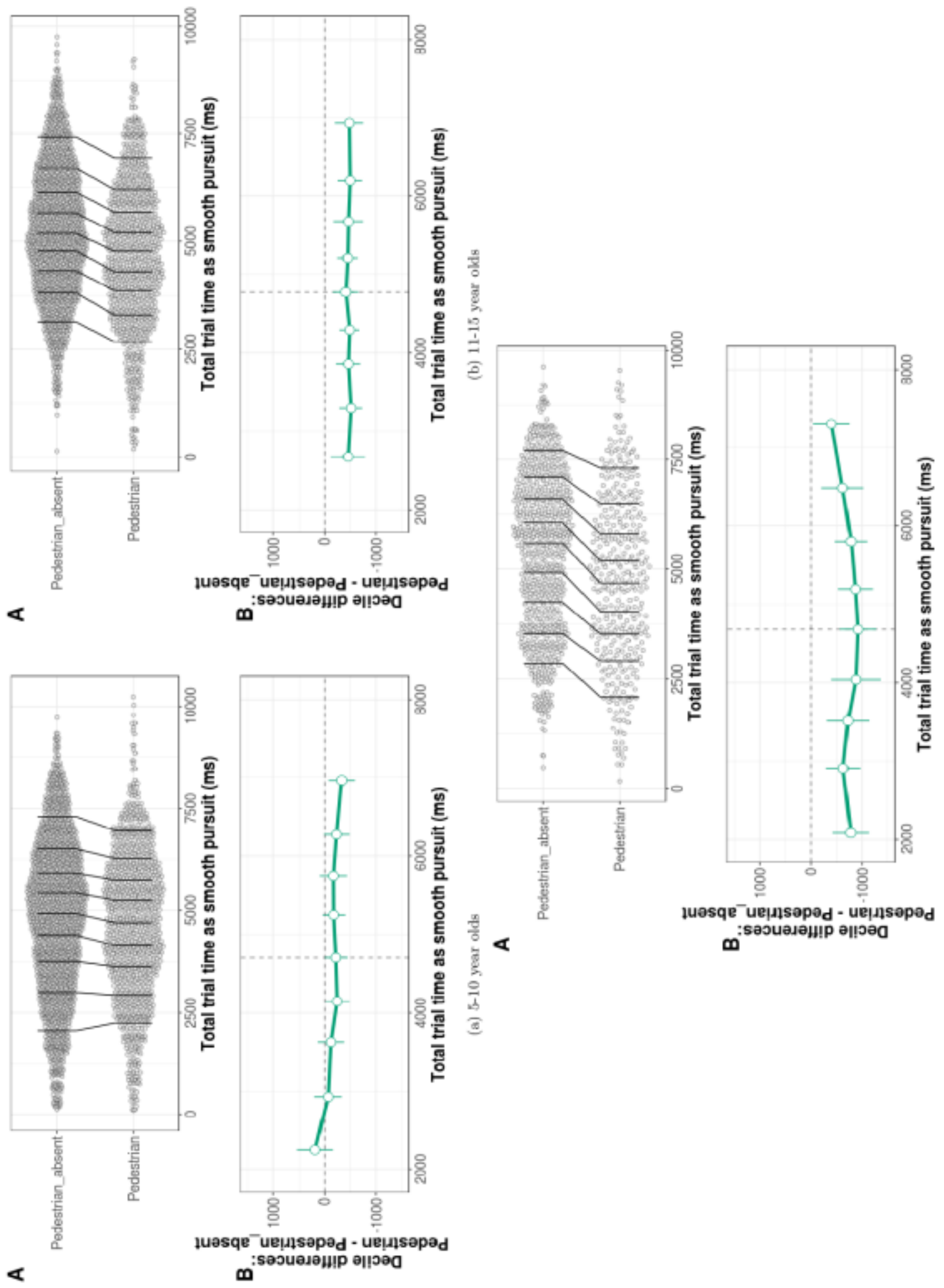

Figure S9. Shift functions for the proportion of trial time as smooth pursuit with pedestrian presence (a) shows the difference in the proportion of trial time as smooth pursuit for 5-10y/os, (b) for 11-15y/os, and (c) for adults on trials where pedestrians are present and trials where pedestrians are not present.

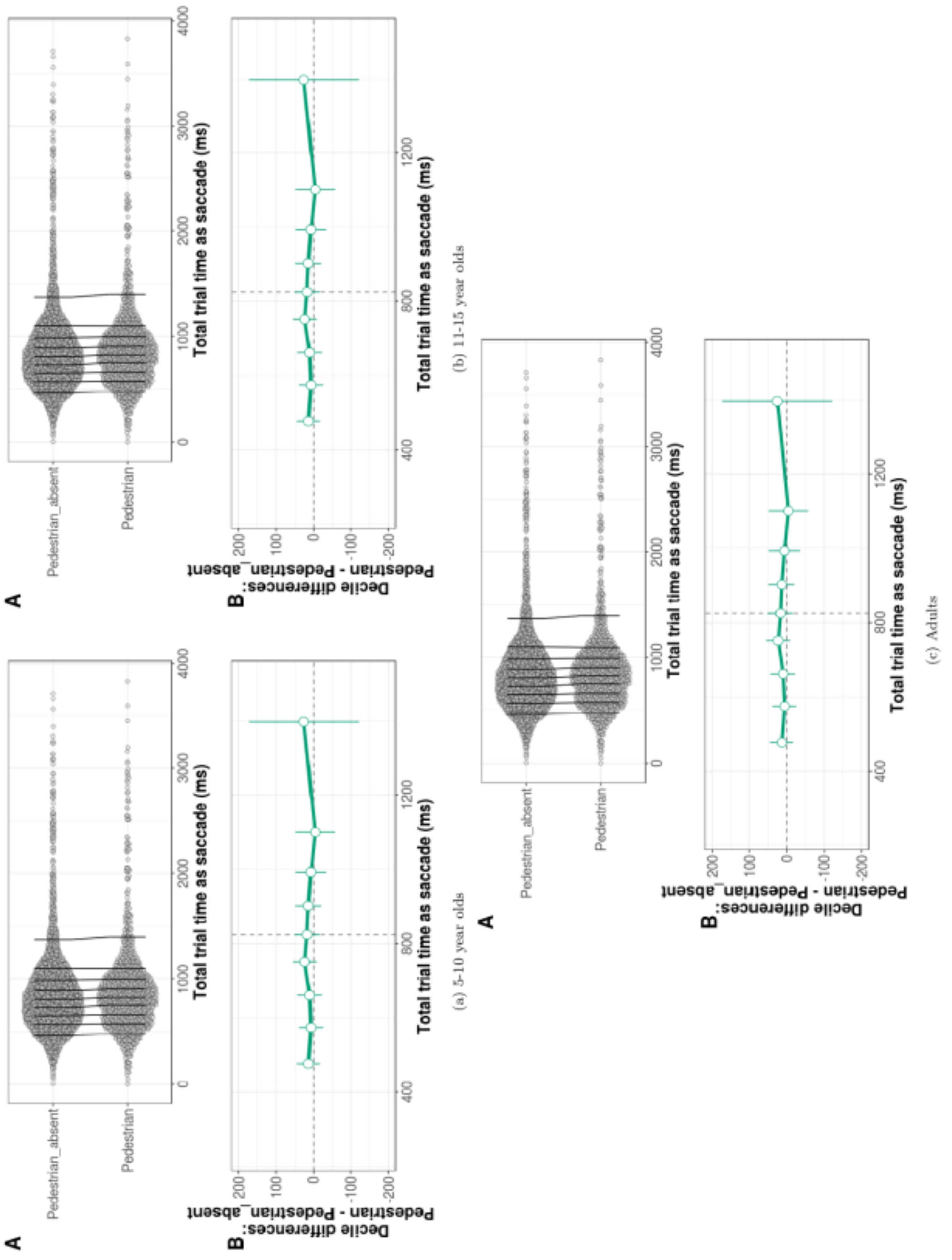

Figure S10. Shift functions for the proportion of trial time as saccades with pedestrian presence (a) shows the difference in the proportion of trial time as saccades for 5-10y/os, (b) for 11-15y/os, and (c) for adults on trials where pedestrians are present and trials where pedestrians are not present.

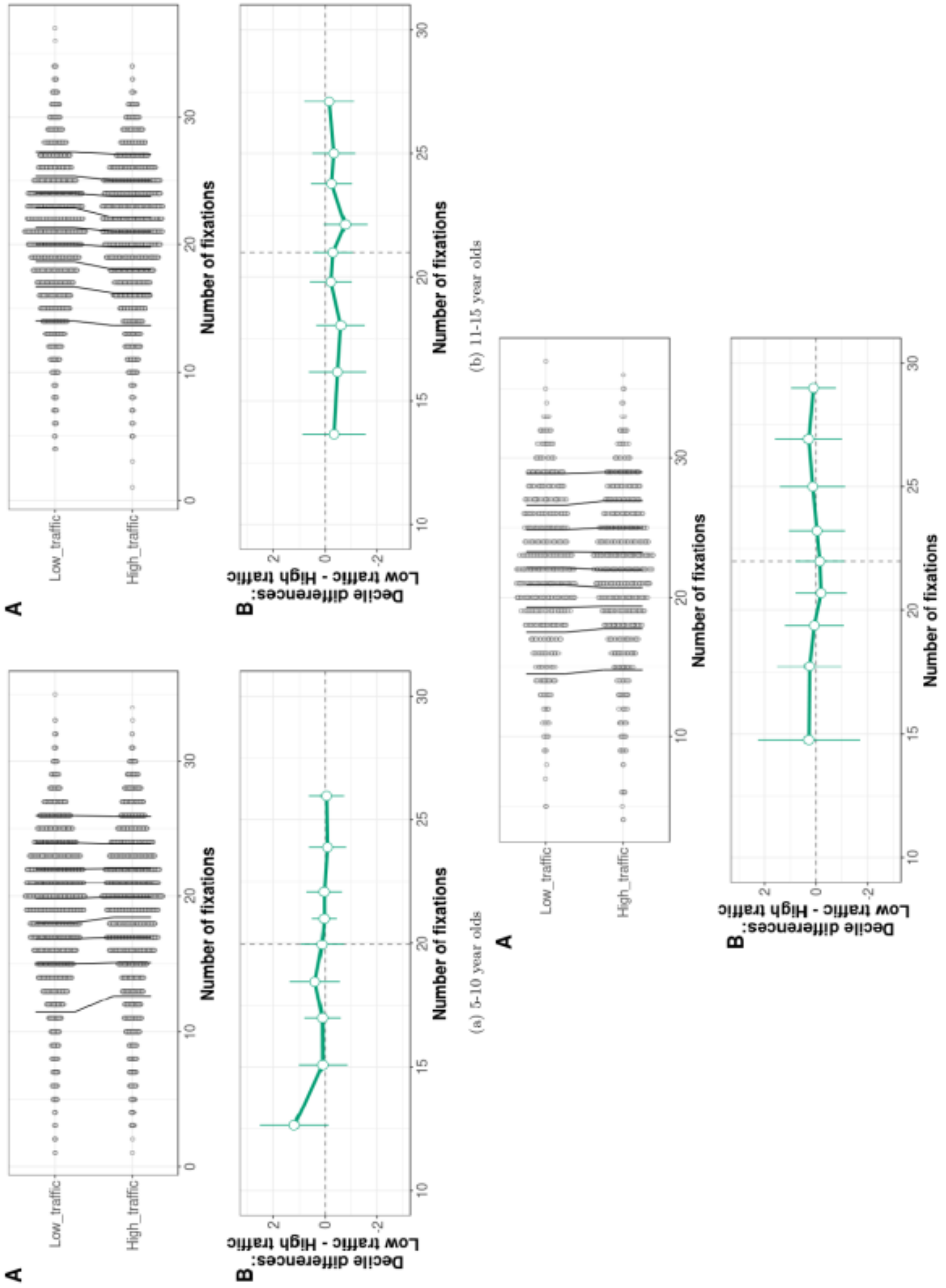

(a) 5-10 year olds

(b) 11-15 year olds

(c) Adults

Figure S11. Shift functions for the number of fixations with traffic density (a) shows the difference in the number of fixations for 5-10y/os, (b) for 11-15y/os, and (c) for adults on trials with low and high traffic density.

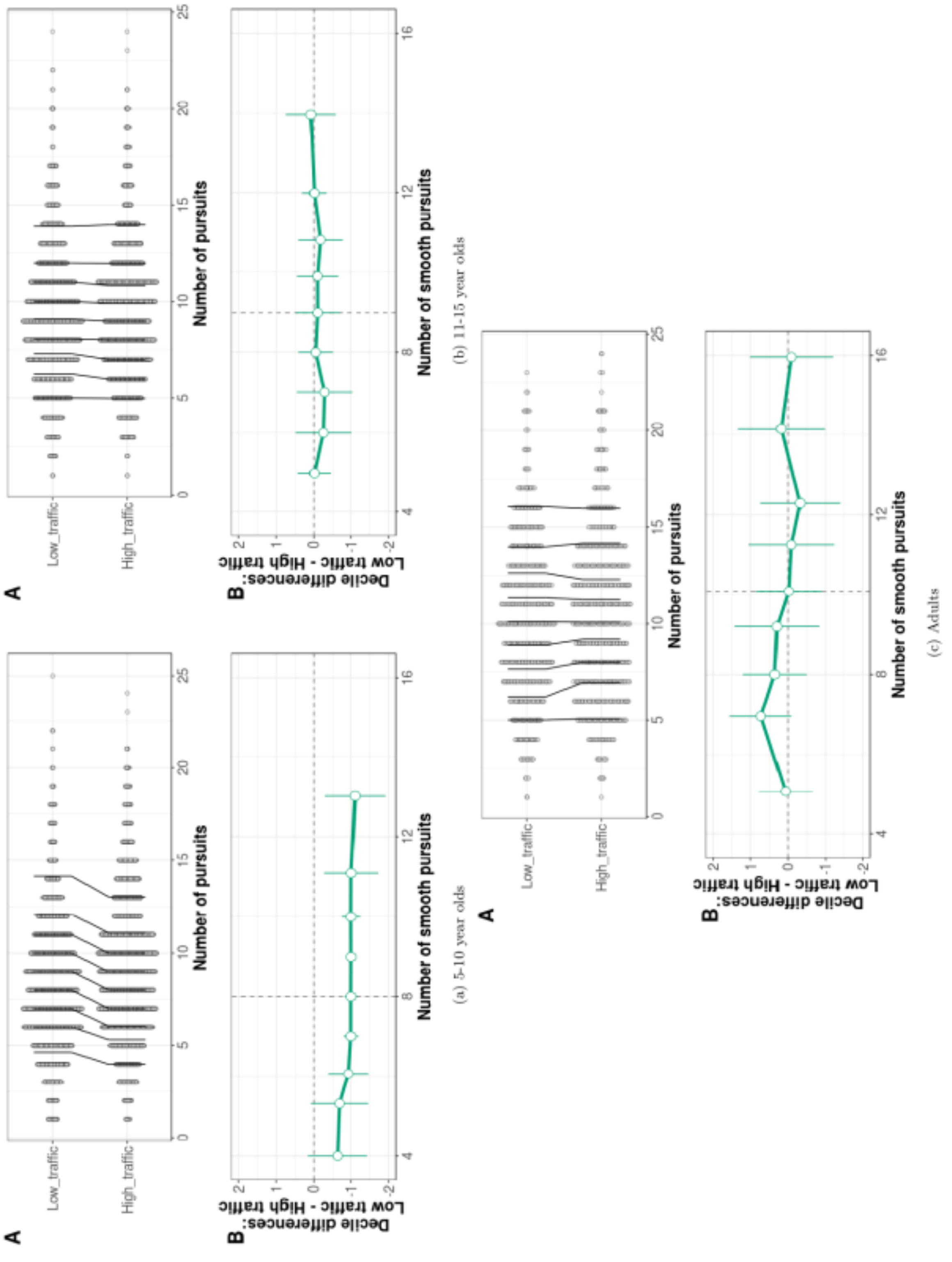

Figure S12. Shift functions for the number of smooth pursuits with traffic density (a) shows the difference in the number of smooth pursuits for 5-10y/os, (b) for 11-15 y/os, and (c) for adults on trials with low and high traffic density.

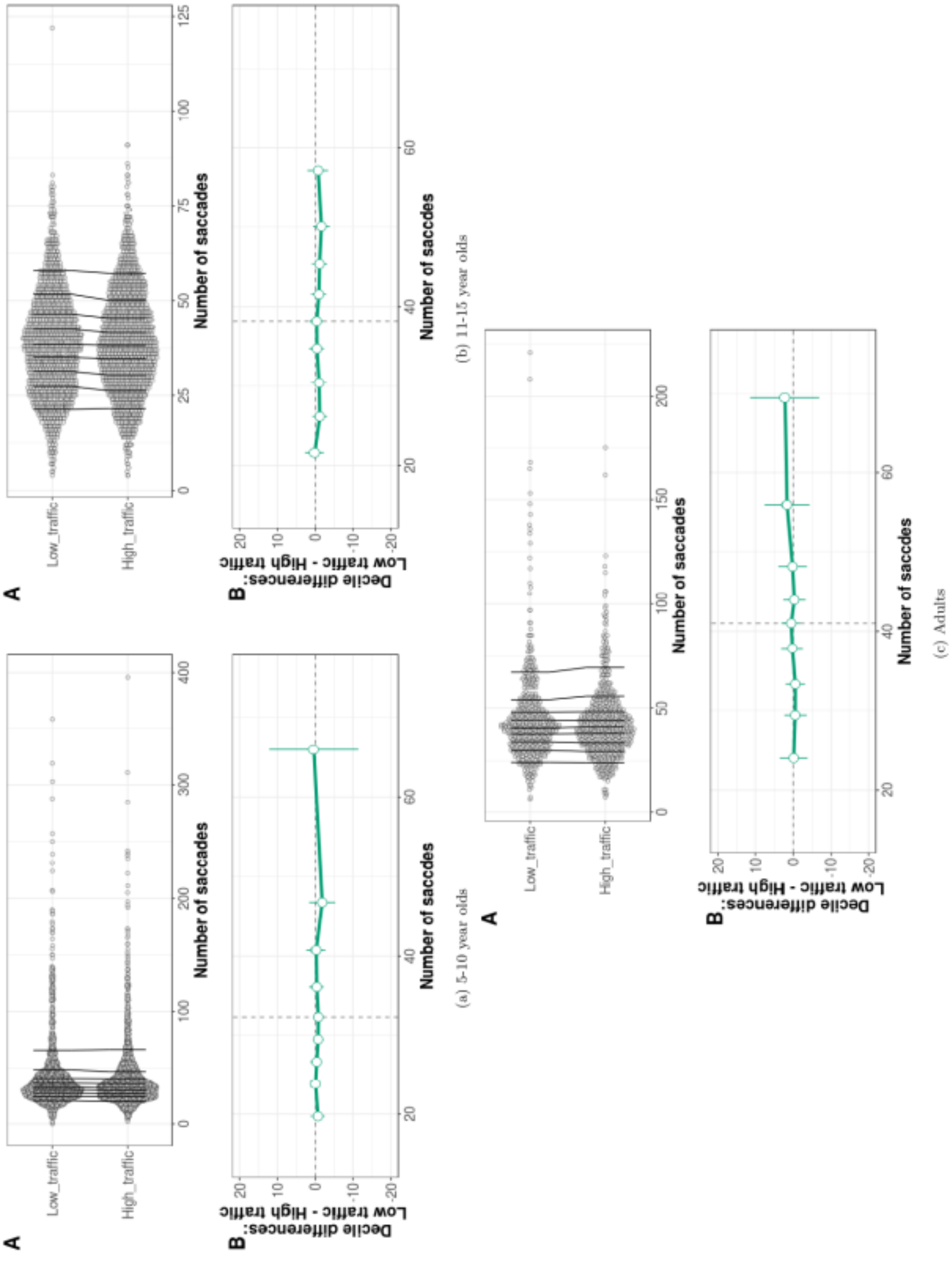

Figure S13. Shift functions for the number of saccades with traffic density (a) shows the difference in the number of saccades for 5-10y/os, (b) for 11-15y/os, and (c) for adults on trials with low and high traffic density.

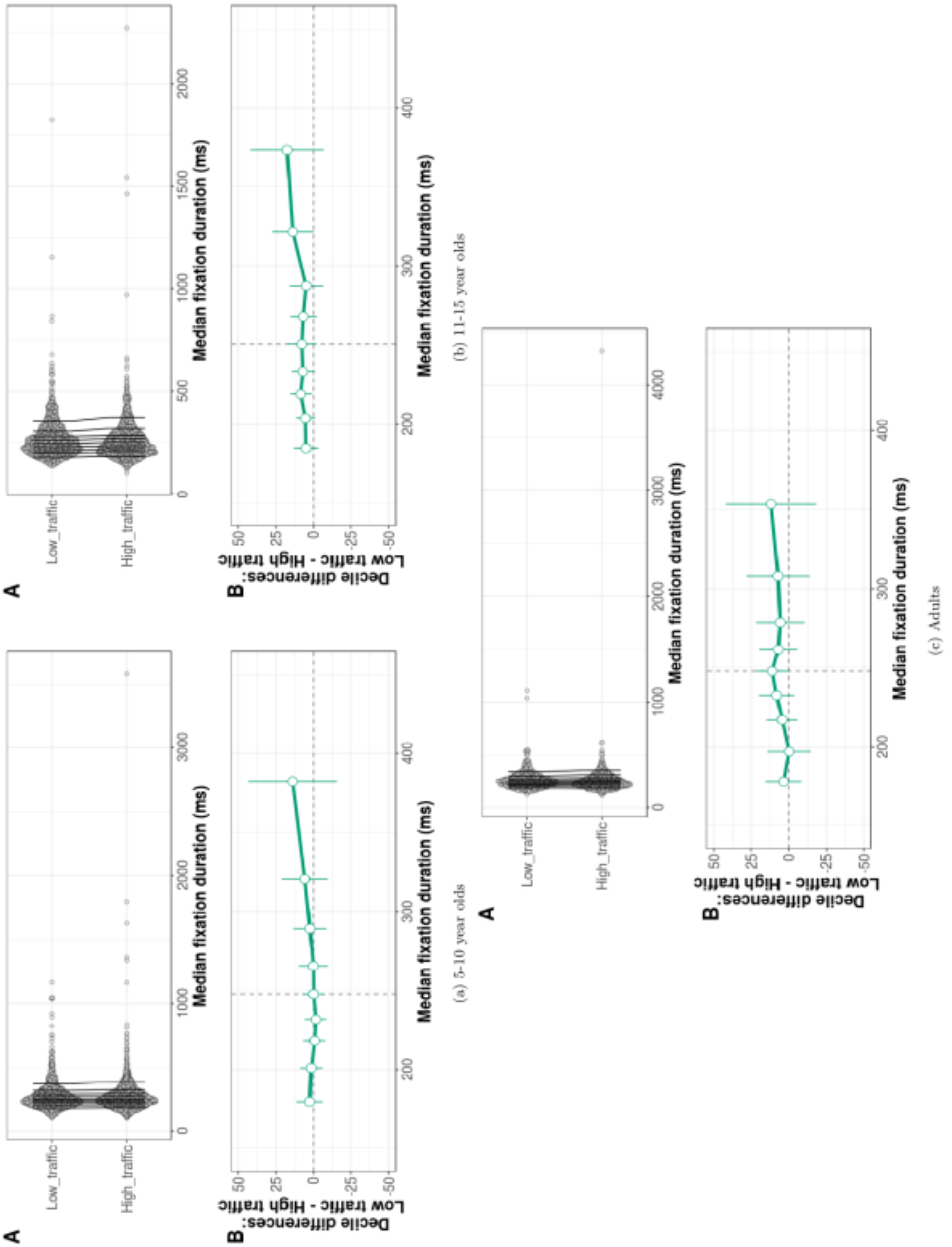

Figure S14. Shift functions for the median fixation duration for traffic density (a) shows the difference in the median fixation duration for 5-10y/os, (b) for 11-15y/os, and (c) for adults on trials with low and high traffic density.

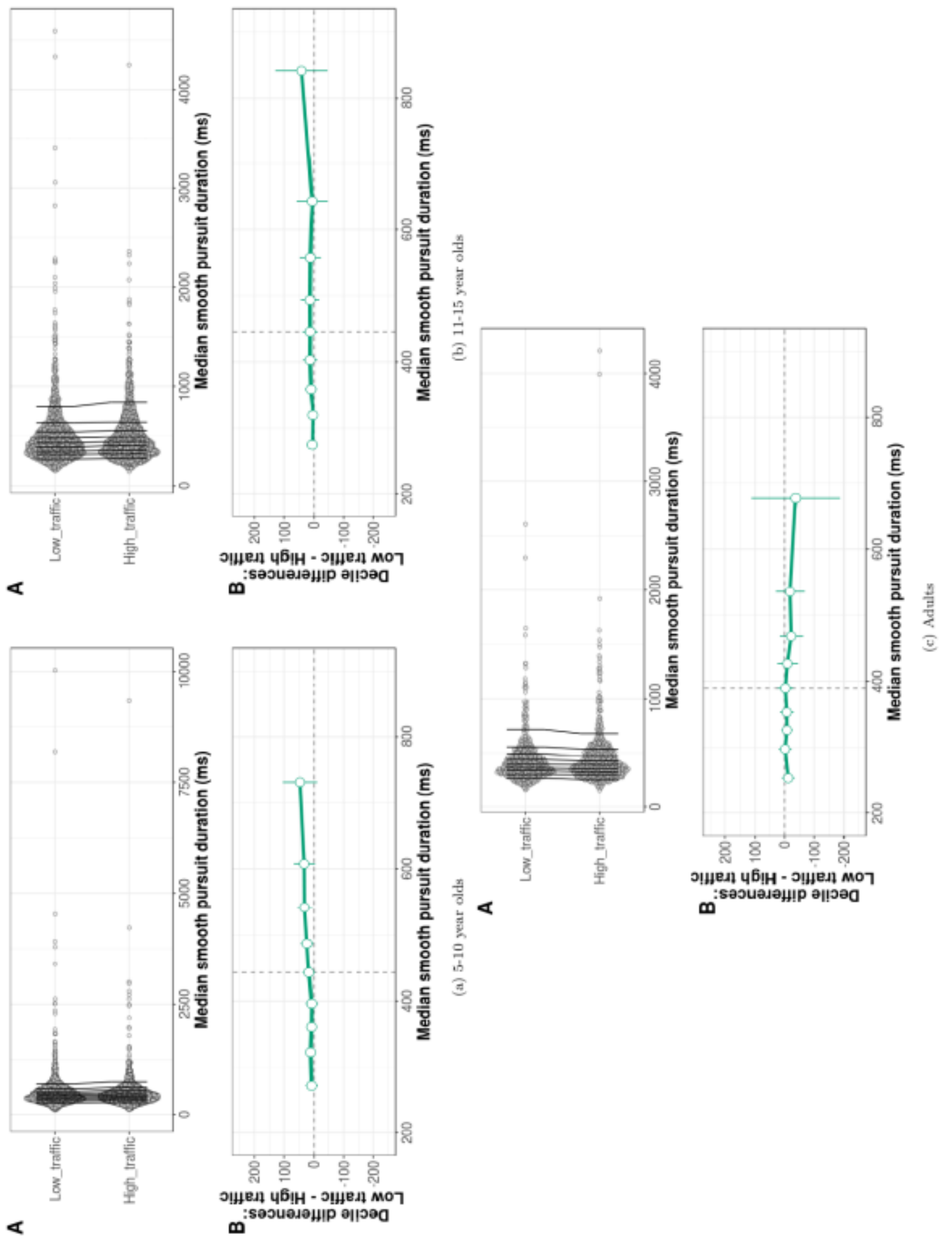

Figure S15. Shift functions for the median smooth pursuit duration for traffic density (a) shows the difference in the median smooth pursuit duration for 5-10y/os, (b) for 11-15y/os, and (c) for adults on trials with low and high traffic density.

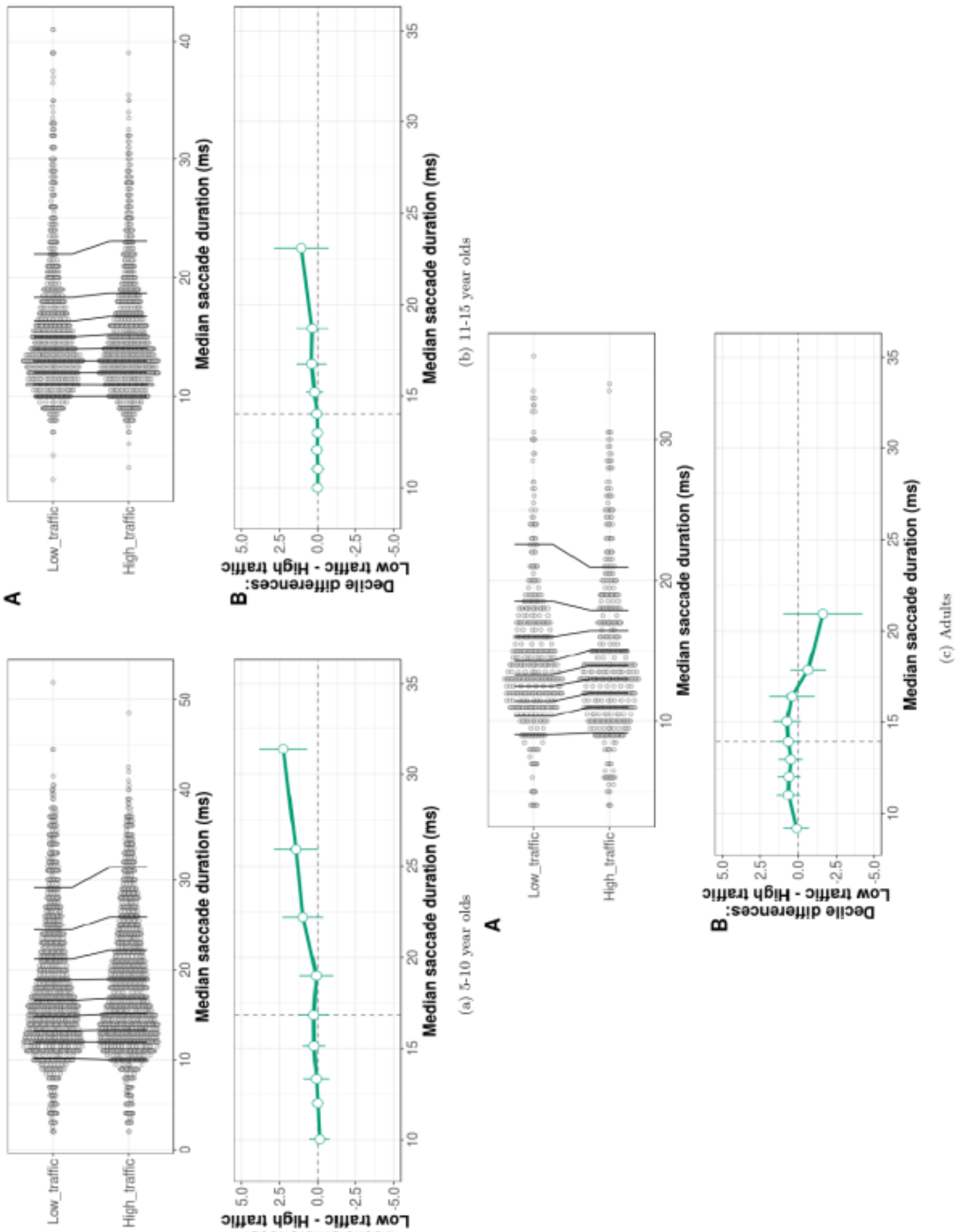

Figure S16. Shift functions for the median saccade duration for traffic density (a) shows the difference in the median saccade duration for 5-10y/os, (b) for 11-15y/os, and (c) for adults on trials with low and high traffic density.

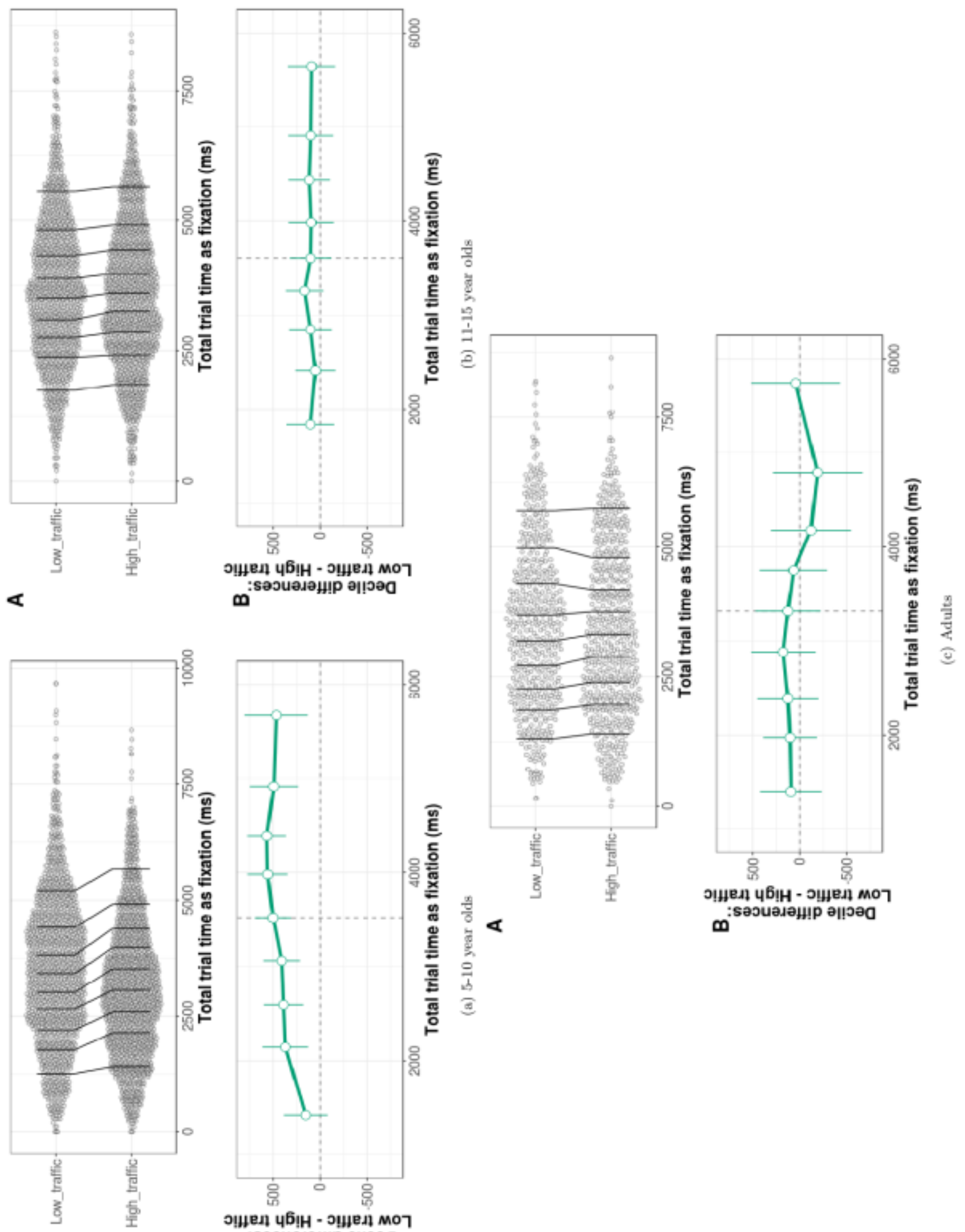

Figure S17. Shift functions for the proportion of trial time as fixation with traffic density (a) shows the difference in the proportion of trial time as fixation for 5-10y/os, (b) for 11-15y/os, and (c) for adults on trials with low and high traffic density.

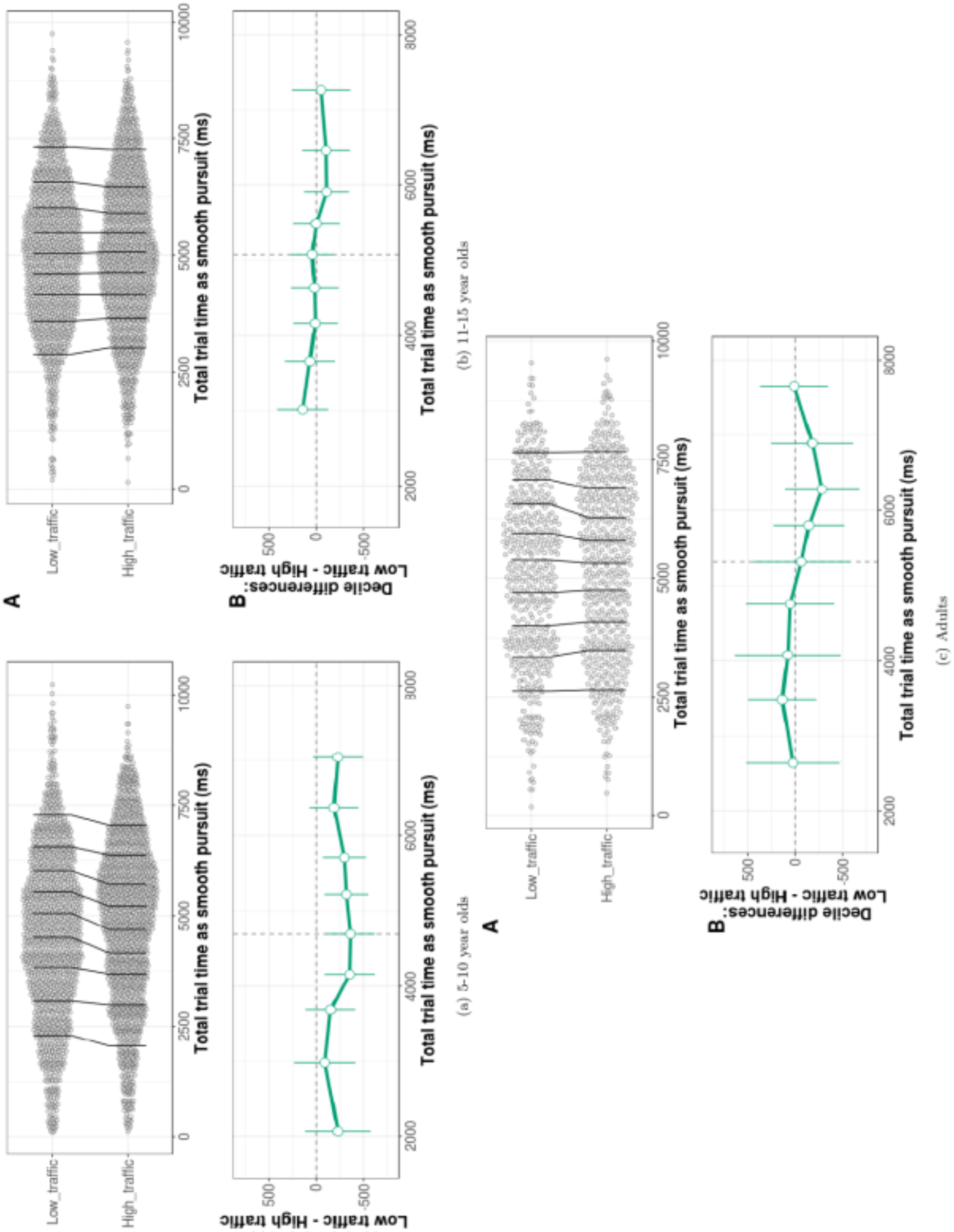

Figure S18. Shift functions for the proportion of trial time as smooth pursuit with traffic density (a) shows the difference in the proportion of trial time as smooth pursuit for 5-10y/os, (b) for 11-15y/os, and (c) for adults on trials with low and high traffic density.

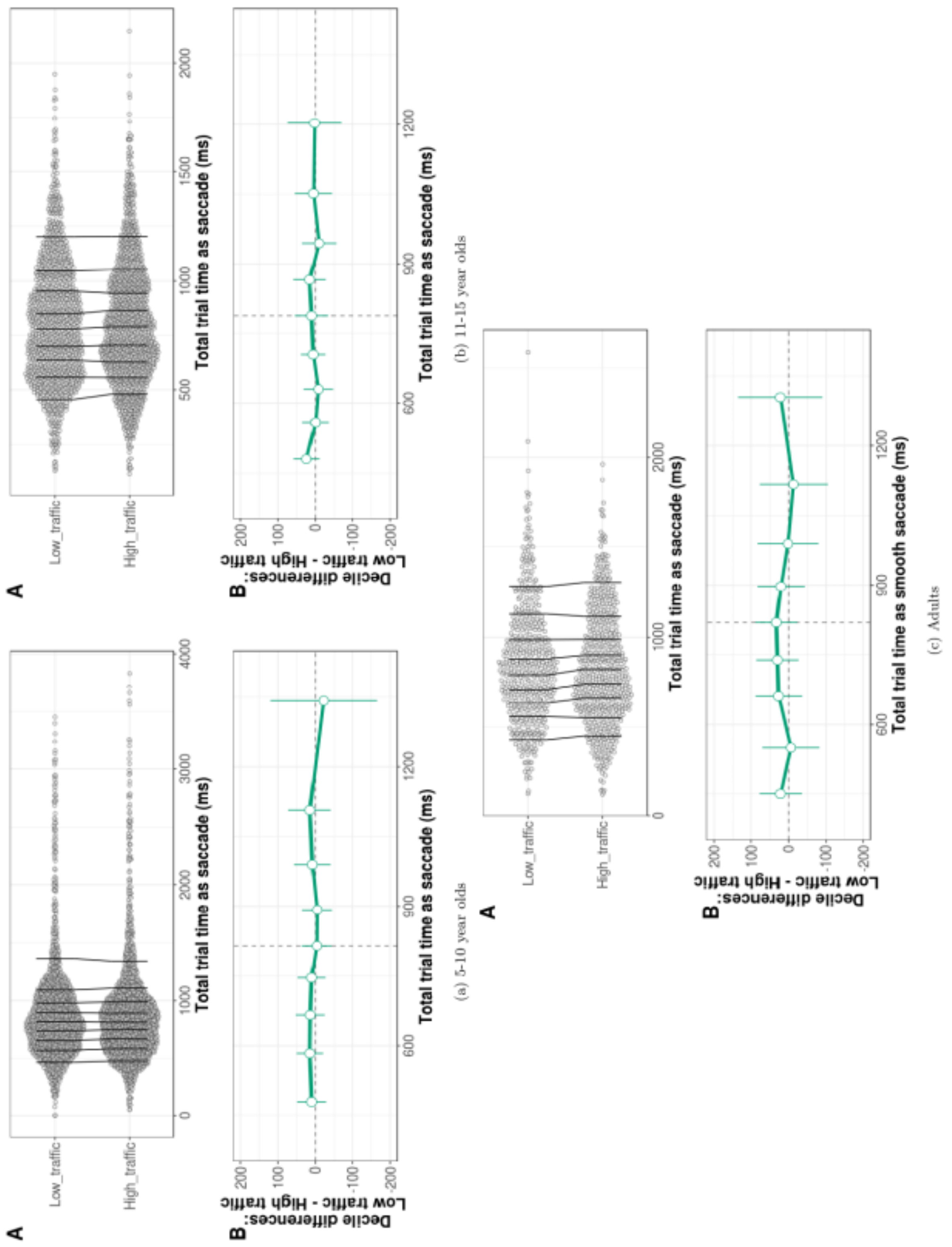

Figure S19. Shift functions for the proportion of trial time as saccades with traffic density (a) shows the difference in the proportion of trial time as saccade for 5-10y/os, (b) for 11-15y/os, and (c) for adults on trials with low and high traffic density.

S20. Summary of the shift function analysis

5-10y/os made significantly more fixations (complete shift) when human distractors were present in the trial (Fig. S2a in the Supplemental Material). On the majority of trials where pedestrians were present 11-15y/os made significantly more fixations. This effect was driven by trials where there were few, and trials where there were many fixations as deciles in the centre of the distribution were not significantly different from each other; though the median did show a significant difference – decile 5 (Fig, S2b in the Supplemental Material).

11-15y/os and adults made significantly fewer pursuits when human distractors were present – although the difference decreased in amplitude on trials with larger number of pursuits to a point where trials with the largest number of pursuits did not show a significant difference (Fig. S3b,c in the Supplemental Material).

11-15y/os and adults had a significantly larger proportion of trial time as fixation (complete shift) when pedestrians were present (Fig. S8b,c in the Supplemental Material). For 5-10y/os, trials which had a medium and large proportion of trial time as fixation were significantly affected by pedestrian presence. On trials where the proportion of trial time as fixation was small there was no significant effect of pedestrian presence (Fig. S8a in the Supplemental Material).

11-15y/os and adults had a significantly smaller proportion of trial time as pursuit gaze samples (complete shift) when pedestrians were present (Fig. S9b,c in the Supplemental Material).

5-10y/os made significantly fewer pursuits when traffic density was low, however, for trials with a small number of pursuits the effect of traffic density was not significant (Fig. S12a in the Supplemental Material).

No other consistent effects were found. In sum, all age groups showed an impact of pedestrians on some of their global oculomotor characteristics while only 5-10y/os showed an impact of traffic density.
