# Supplementary figures and images for: Developing attentional control in naturalistic dynamic road crossing situations
Source: Sci Rep. 2019 Mar 12;9:4176. doi: 10.1038/s41598-019-39737-7 (PMC6414534; doi:10.1038/s41598-019-39737-7)

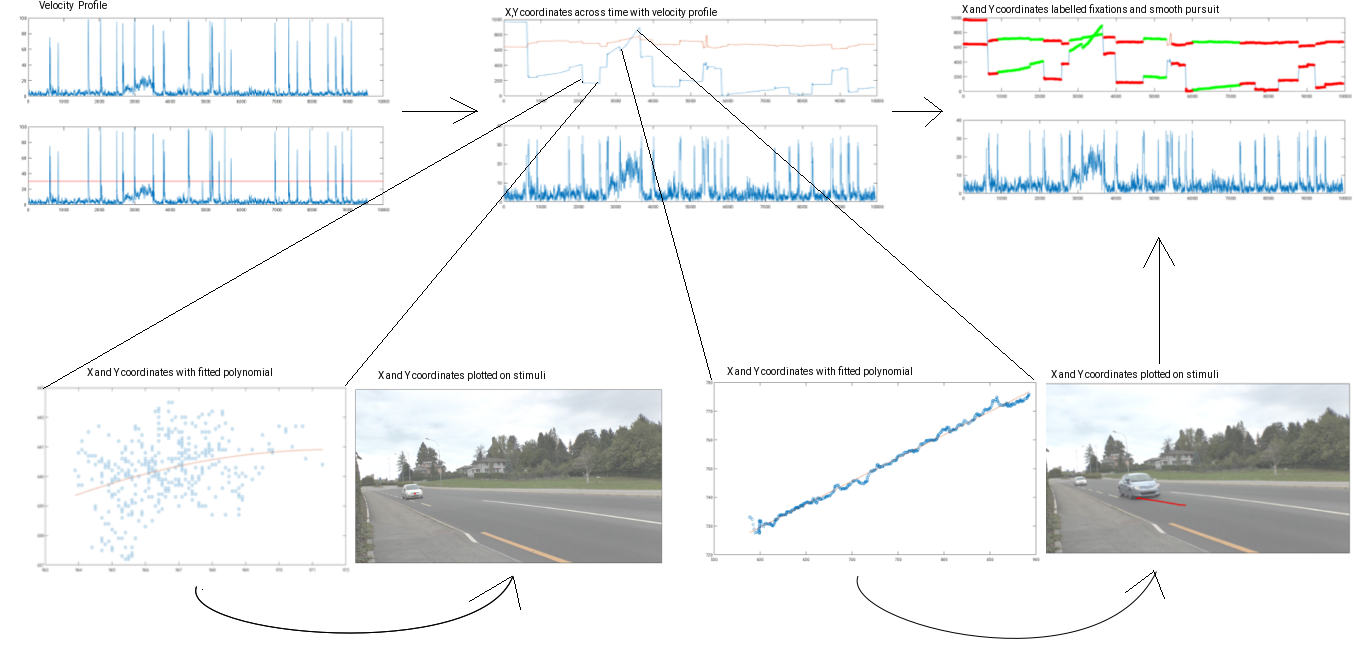

Supplement: Supplementary file 2 — LaTeX Supplementary File [file 41598_2019_39737_MOESM2_ESM.png]

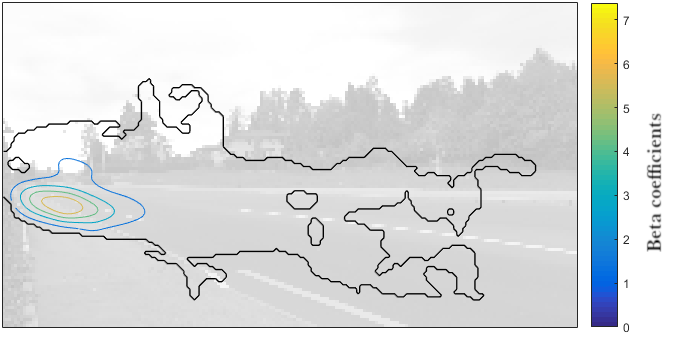

Supplement: Supplementary file 3 — LaTeX Supplementary File [file 41598_2019_39737_MOESM3_ESM.png]

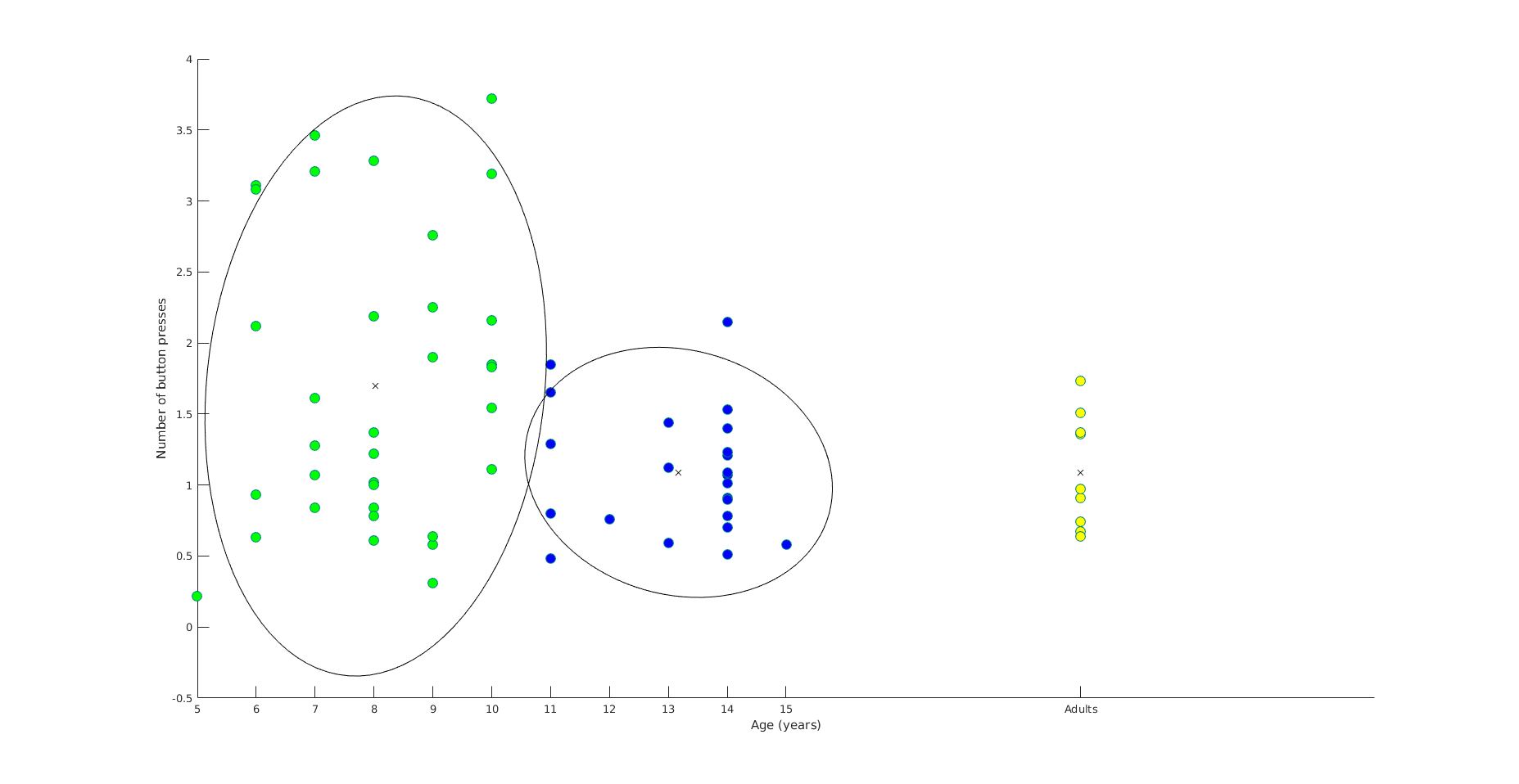

Supplement: Supplementary file 4 — LaTeX Supplementary File [file 41598_2019_39737_MOESM4_ESM.png]

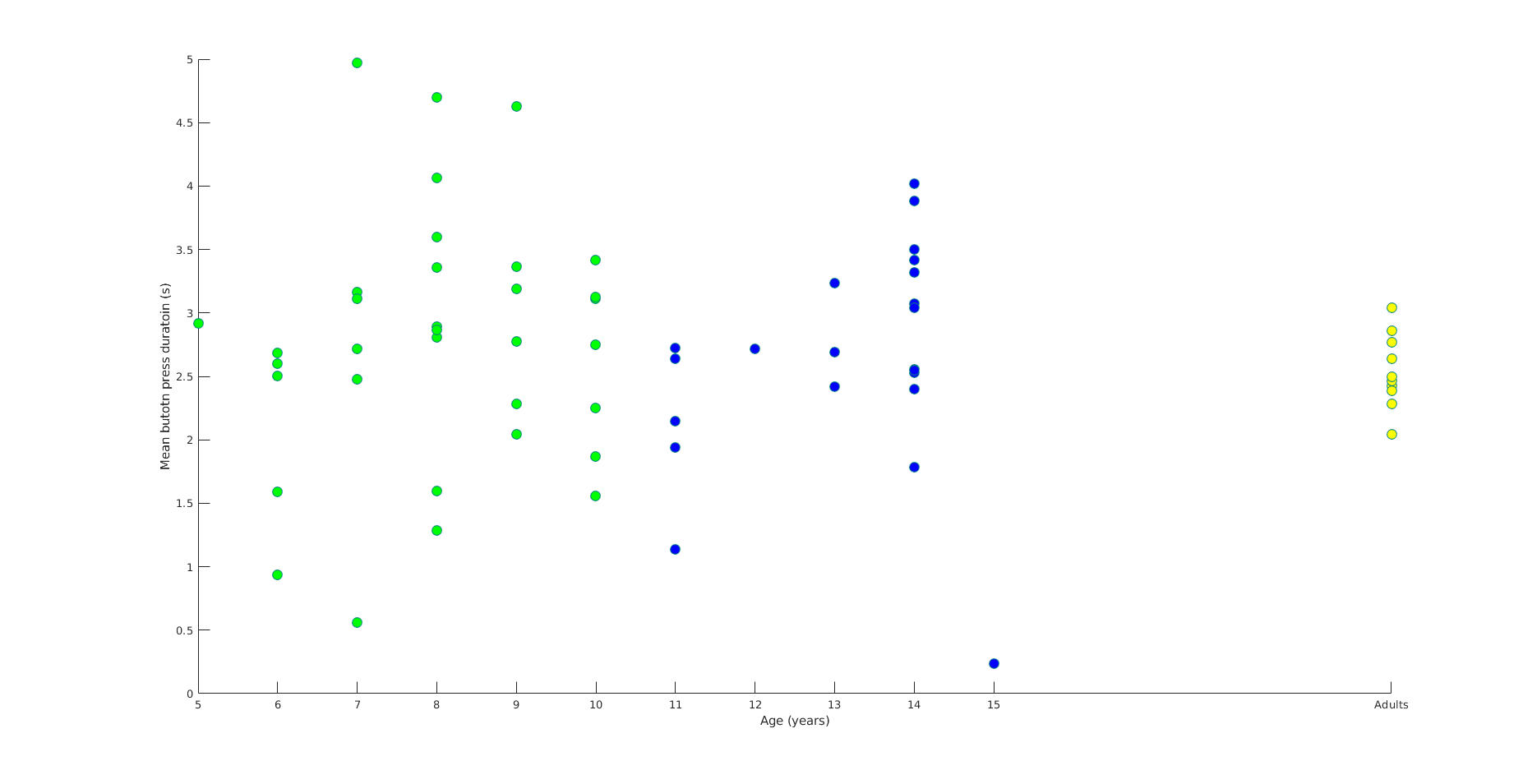

Supplement: Supplementary file 5 — LaTeX Supplementary File [file 41598_2019_39737_MOESM5_ESM.png]

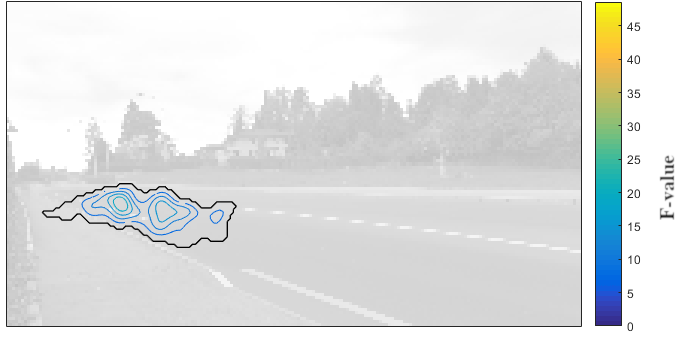

Supplement: Supplementary file 6 — LaTeX Supplementary File [file 41598_2019_39737_MOESM6_ESM.png]

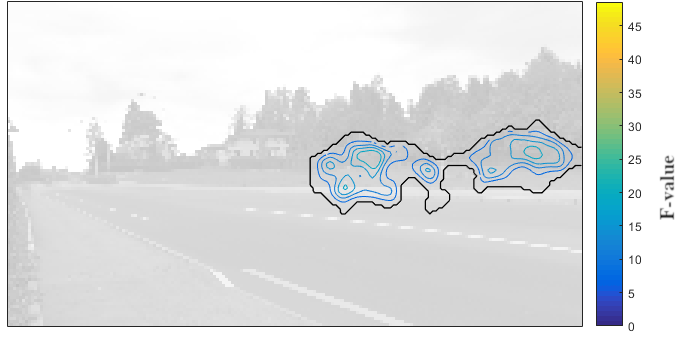

Supplement: Supplementary file 7 — LaTeX Supplementary File [file 41598_2019_39737_MOESM7_ESM.png]

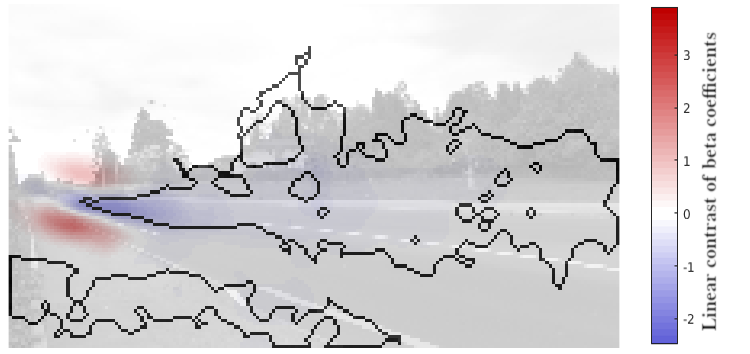

Supplement: Supplementary file 8 — LaTeX Supplementary File [file 41598_2019_39737_MOESM8_ESM.png]

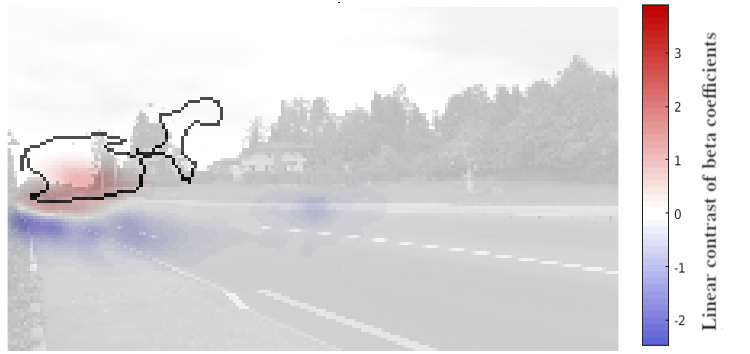

Supplement: Supplementary file 9 — LaTeX Supplementary File [file 41598_2019_39737_MOESM9_ESM.png]

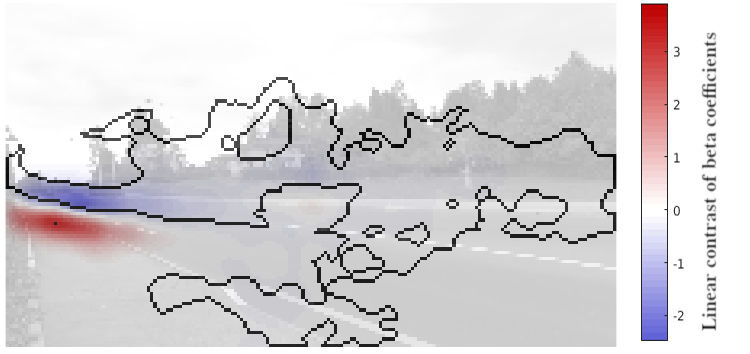

Supplement: Supplementary file 10 — LaTeX Supplementary File [file 41598_2019_39737_MOESM10_ESM.png]

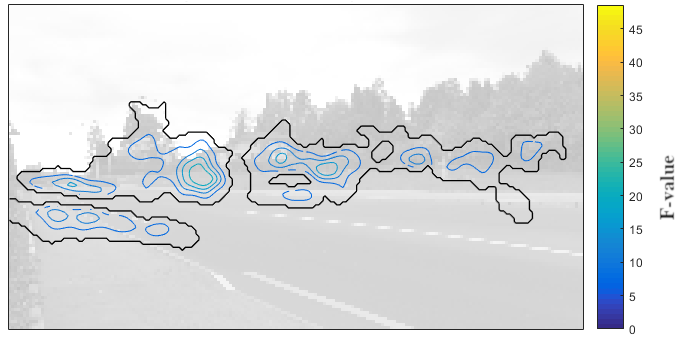

Supplement: Supplementary file 11 — LaTeX Supplementary File [file 41598_2019_39737_MOESM11_ESM.png]

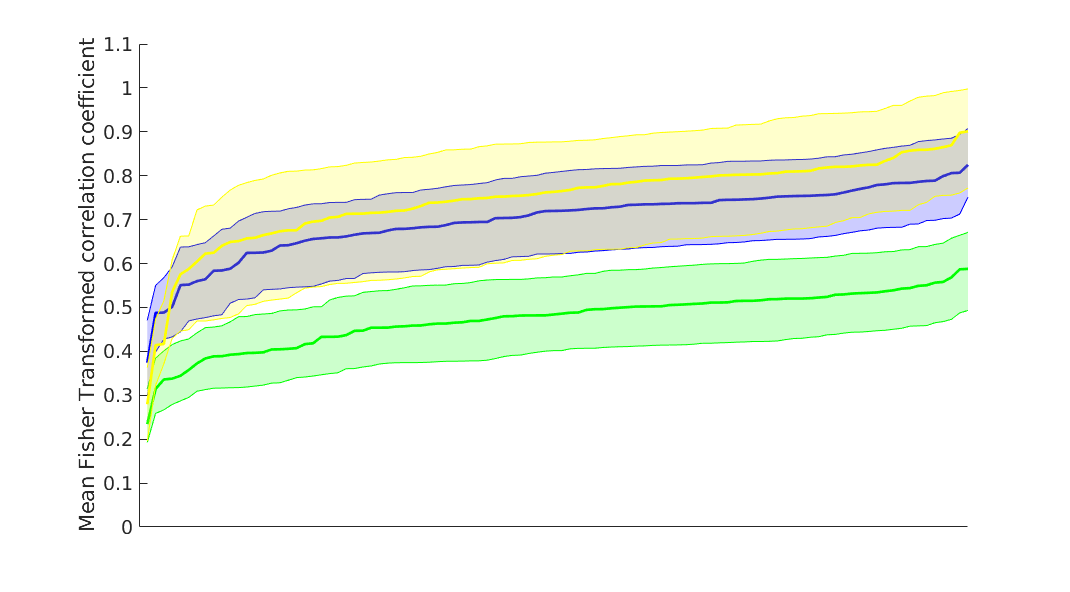

Supplement: Supplementary file 12 — LaTeX Supplementary File [file 41598_2019_39737_MOESM12_ESM.png]

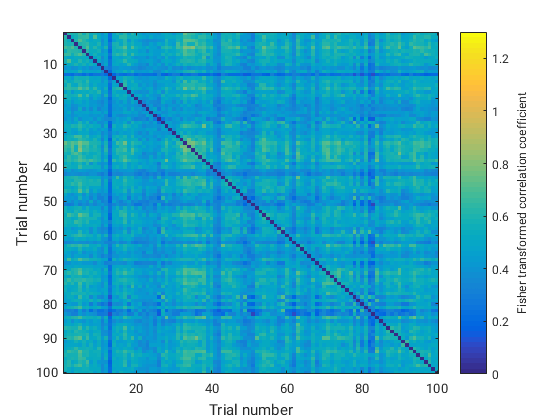

Supplement: Supplementary file 13 — LaTeX Supplementary File [file 41598_2019_39737_MOESM13_ESM.png]

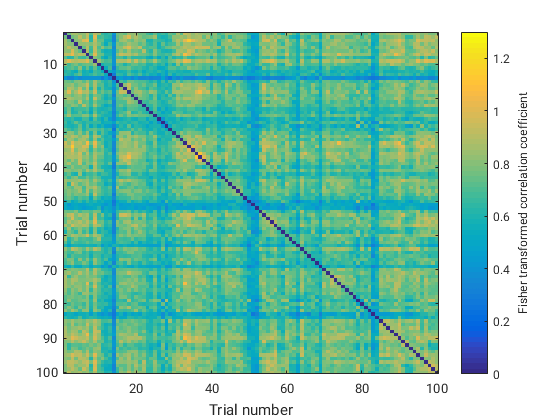

Supplement: Supplementary file 14 — LaTeX Supplementary File [file 41598_2019_39737_MOESM14_ESM.png]

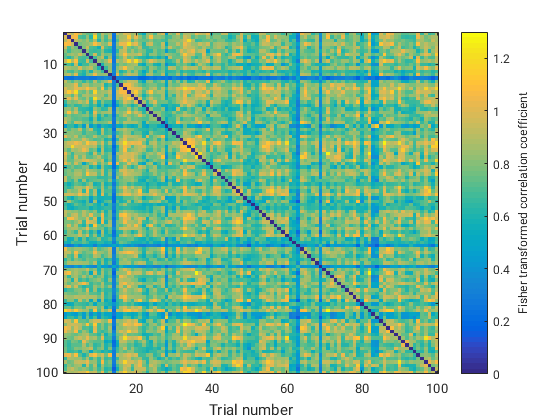

Supplement: Supplementary file 15 — LaTeX Supplementary File [file 41598_2019_39737_MOESM15_ESM.png]

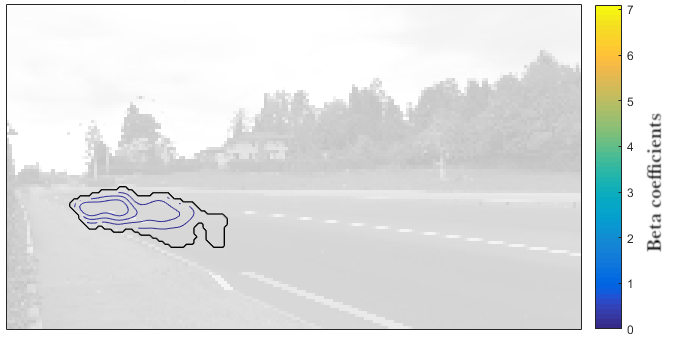

Supplement: Supplementary file 16 — LaTeX Supplementary File [file 41598_2019_39737_MOESM16_ESM.png]

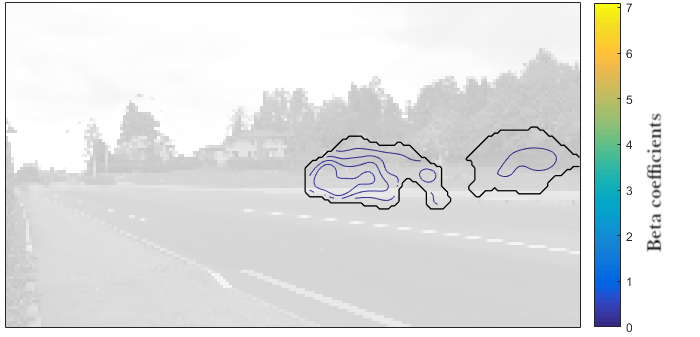

Supplement: Supplementary file 17 — LaTeX Supplementary File [file 41598_2019_39737_MOESM17_ESM.png]

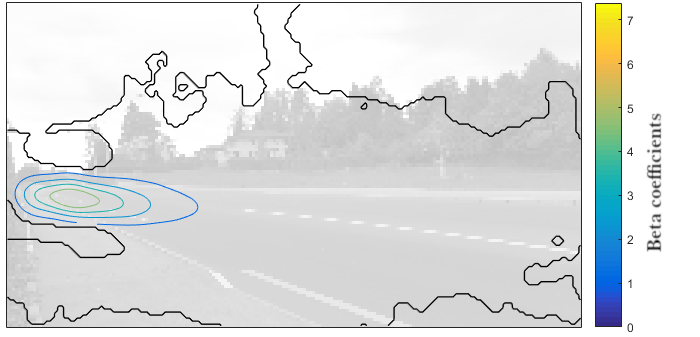

Supplement: Supplementary file 18 — LaTeX Supplementary File [file 41598_2019_39737_MOESM18_ESM.png]

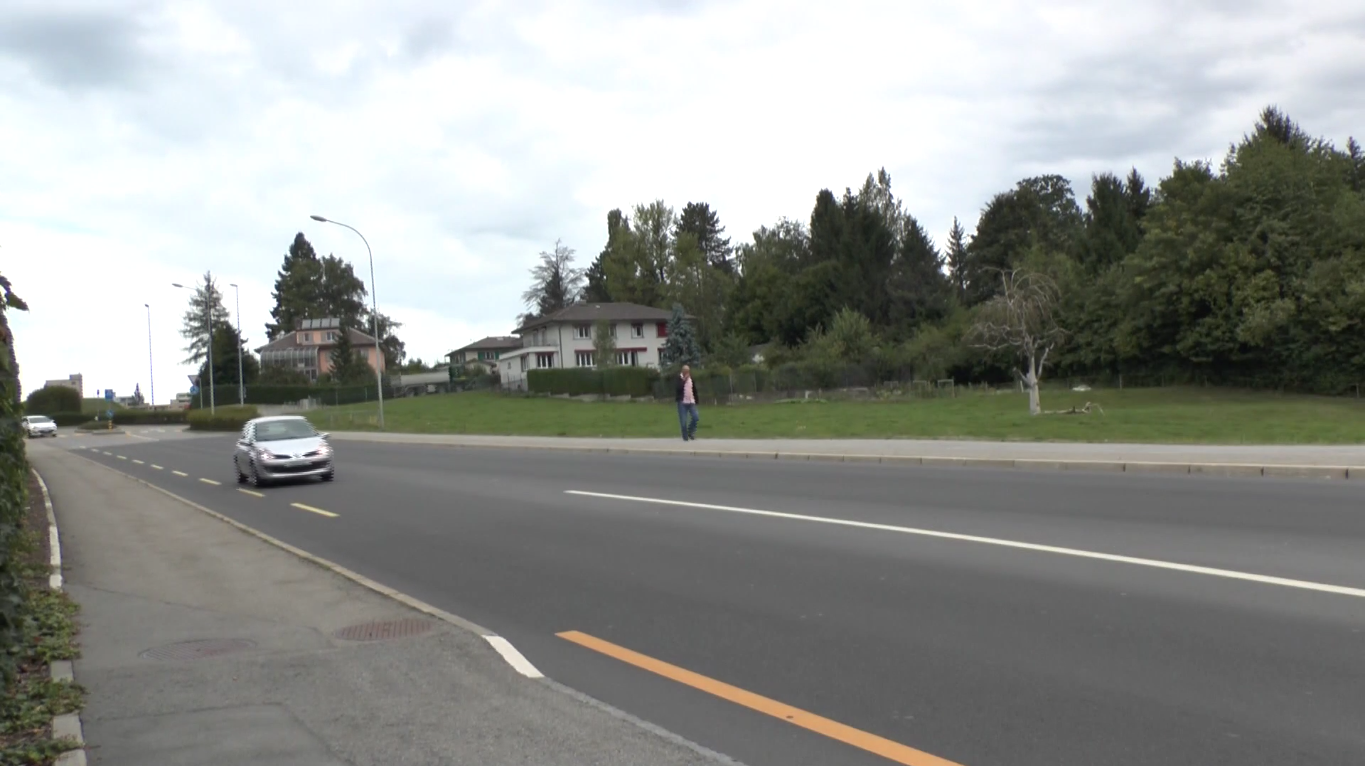

Supplement: Supplementary file 19 — LaTeX Supplementary File [file 41598_2019_39737_MOESM19_ESM.png]

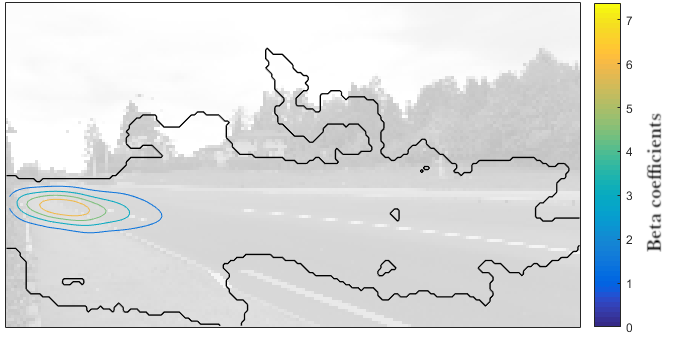

Supplement: Supplementary file 20 — LaTeX Supplementary File [file 41598_2019_39737_MOESM20_ESM.png]
